# Unexpected relationships between frequency of antimicrobial resistance, disease phenotype and *emm* type in group A *Streptococcus*

**DOI:** 10.1099/mgen.0.000316

**Published:** 2019-11-22

**Authors:** Misu A. Sanson, Olga R. Macias, Brittany J. Shah, Blake Hanson, Luis Alberto Vega, Zain Alamarat, Anthony R. Flores

**Affiliations:** ^1^​ Division of Infectious Diseases, Department of Pediatrics, McGovern Medical School, Houston, TX, USA; ^2^​ Center for Antimicrobial Resistance and Microbial Genomics, University of Texas Health Sciences Center at Houston, Houston, TX, USA

**Keywords:** group A *Streptococcus*, invasive, skin and soft tissue, pharyngitis, antimicrobial resistance, *emm *type

## Abstract

Despite universal susceptibility to β-lactams, resistance to second-line antimicrobials (e.g. erythromycin) is increasingly common among group A *Streptococcus* (GAS). To better understand the frequency of regional GAS antimicrobial resistance, we screened a previously described GAS strain collection from Houston, TX, USA, for resistance to commonly used antimicrobials. A total of 100/929 (10.8 %) showed resistance to at least one antimicrobial. Tetracycline resistance was identified in 52 (5.6 %) GAS strains. The cumulative frequency of erythromycin and clindamycin resistance [macrolide (M) and macrolide-lincosamide-streptogramin (MLS) phenotypes] was greatest among invasive GAS strains (9.9 %) compared to that of strains derived from any other infection type (5.9 %, *P*=0.045). We identified *emm* types 11, 75, 77 and 92 as the only *emm* types with high (e.g. >50 %) within-*emm* type resistance and contributing to the majority (24/26; 92 %) of erythromycin/clindamycin resistance in invasive GAS. High-frequency resistance *emm* types were also significantly overrepresented in invasive GAS strains as indicated by invasive index. We performed whole-genome sequencing to define genetic elements associated with resistance among *emm* types 11, 75, 77 and 92. Diverse mobile elements contributed to GAS resistance including transposons, integrative conjugative elements, prophage and a plasmid. Phylogenetic analysis suggests recent clonal emergence of *emm92* GAS strains. Our findings indicate that less frequently encountered GAS *emm* types disproportionately contribute to resistance phenotypes, are defined by diverse mobile genetic elements and may favour invasive disease.

## Data Summary

All raw genome sequences generated in this study are publicly available from the National Center for Biotechnology Information (NCBI) under BioProject accession number PRJNA494557. Comparative phylogenomics analysis was conducted using publicly available data from the NCBI under BioProject numbers PRJNA395240 and PRJEB13551, as described in Methods, and the data are listed in Data Bibliography. The programs used to analyse raw sequence reads, for polymorphism discovery, and whole-genome sequence based phylogenies are available for download as described in the Supplementary Material. The CLC Genomics Workbench is available for purchase at www.qiagenbioinformatics.com

**Impact Statement**

Group A *Streptococcus* (GAS) is a human pathogen responsible for a wide range of diseases. GAS strains are universally susceptible to penicillin, with no history of resistance to this class of antibiotics. However, use of second-line agents such as macrolides (e.g. erythromycin) has increased dramatically for relatively benign conditions, such as pharyngitis. Concomitantly, resistance to second-line antimicrobials in GAS has increased to the point that the Centers for Disease Control and Prevention considers macrolide resistance in GAS a ‘concerning level’ threat. We sought to examine the frequency of antimicrobial resistance among paediatric GAS strains collected in Houston, TX, USA. While overall frequency of antimicrobial resistance was low (10 %), we observed a significant association between antimicrobial resistance for any class of antibiotic and invasive disease. Despite constituting only 7 % of the GAS strain population, GAS strains of *emm* types 11, 75, 77 and 92 were responsible for the majority of observed resistance. Whole-genome sequencing of resistant strains revealed that a variety of mobile genetic elements contributed to the observed antimicrobial-resistance phenotypes. Our findings of a correlation between high-frequency antimicrobial resistance encoded in mobile genetic elements and GAS invasive disease warrant continued surveillance, and further investigation of the relationship between antimicrobial resistance and strain virulence in GAS.

## Introduction

*Streptococcus pyogenes* (group A *Streptococcus *; GAS) is a ubiquitous, obligate, human pathogen capable of causing a wide range of diseases. Most frequently, GAS is responsible for relatively benign diseases, such as pharyngitis or superficial skin infections. However, a small subset of so-called ‘benign’ disease manifestations may develop into more severe, life-threatening diseases, such as toxic shock syndrome and necrotizing fasciitis. Invasive disease occurs at a rate of 3–5 cases/100 000 population in the USA, and displays a bimodal age distribution with peaks in the young (<5 years) and elderly (>65 years) [1]. In addition to disease, GAS may also be carried in the throat in the absence of symptoms and such asymptomatic carriage is most common in children aged 5–15 years [2].

Despite the use of penicillin in humans for over 75 years, GAS strains continue to be universally susceptible to it and, thus, penicillin and penicillin-based antimicrobials remain the recommended therapy for infections caused by GAS. However, in cases of penicillin allergy, second-line antimicrobials such as macrolides (e.g. erythromycin and azithromycin), lincosamides (e.g. clindamycin) and tetracyclines are effective therapies [3]. Due to the high frequency of asymptomatic carriage in children, when eradication of the carrier state is desired, second-line agents have proven to be more effective [3]. Use of macrolides and clindamycin has become more common in many paediatric settings as empiric therapy for skin and soft tissue infection (SSTI) and respiratory infection, and may contribute to increased resistance [4, 5]. The potential for increased second-line agent resistance in GAS has led to the Centers for Disease Control and Prevention (CDC) to identify macrolide-resistant GAS as a potential emerging threat [6].

With respect to antimicrobial-resistance mechanisms, GAS strains share similar traits to other streptococci and staphylococci, including mobile genetic elements that confer resistance. Perhaps most common among them are transposons, specifically Tn*916*, that may carry resistance genes for tetracyclines, macrolides and aminoglycosides [7]. In addition to transposons, GAS may carry prophage, integrative conjugative elements (ICEs) and plasmids that possess any number of antimicrobial-resistance factors [8]. Few studies have examined the mobile elements in diverse GAS strains derived from comprehensive surveillance on a local or national level.

We previously described the molecular epidemiology of GAS disease in children in the Houston, TX, metropolitan area (USA) over the course of 4 years (July 2013–2017) [9]. In that study, 930 GAS isolates were identified by convenience sampling of Texas Children’s Hospital (Houston, TX, USA) patients with a GAS-positive culture result. In this study, we screened those GAS isolates for resistance to common antimicrobials used in paediatrics to better understand the frequency of resistance, association of resistance phenotypes to GAS *emm* and disease types, and genetic elements associated with resistance.

## Methods

### Description of the GAS strain collection in Houston, TX

The description of the 930 GAS isolates, disease definitions and methods, including molecular typing, have been previously published [9]. Isolates were collected under a protocol approved by the Committee for the Protection of Human Subjects at the University of Texas Health Sciences Center (Houston, USA). One isolate was not tested for antimicrobial susceptibility due to non-viability. Very briefly, GAS strains were collected from leftover patient samples with a GAS-positive culture result by the clinical microbiology laboratory at the Texas Children’s Hospital from July 1 2013 to June 30 2017. Strains were *emm* typed and assigned a disease category of invasive (*n*=263), SSTI (*n*=235) or pharyngeal (*n*=431), excluding isolates recovered from asymptomatic cases as previously described [9].

### Bacterial growth and susceptibility testing

GAS strains were grown using trypticase soy agar supplemented with 5 % sheep blood (SBA), in Todd–Hewitt broth supplemented with 0.2 % yeast extract (THY) or on THY agar. The Clinical and Laboratory Standards Institute (CLSI) protocol was followed for antimicrobial disc diffusion susceptibility testing [10]. Strains were grown on BD BBL Mueller–Hinton agar with 5 % sheep blood at 37 °C supplemented with 5 % CO_2_. Zones of inhibition were measured using digital callipers following a minimum of 18 h of growth. Interpretations (susceptible, intermediate or resistant) were made using CLSI breakpoints for β-haemolytic streptococci [10]. Antimicrobial discs (BD Sensi-Disc; ThermoFisher) included chloramphenicol (30 µg), clindamycin (2 µg), erythromycin (15 µg), levofloxacin (5 µg), penicillin (10 U) and tetracycline (30 µg). Clindamycin and erythromycin discs were placed adjacent to each other to identify inducible resistance to clindamycin (D-test). Resistance phenotypes to erythromycin, clindamycin and tetracycline were confirmed using an Etest (bioMérieux). Minimum inhibitory concentrations (MICs) to aminoglycosides (streptomycin, kanamycin and gentamicin) were determined in *emm11* and *emm92* strains using broth microdilution or an Etest. For broth microdilution, strains were grown to an OD_600_ 1.0 in THY broth, diluted 1 : 50 in fresh THY broth, serially diluted with the desired antimicrobial in a 96-well plate, and growth monitored using a BioTek Synergy H1 plate reader. Strains were prepared for Etest as described above for disc diffusion.

### Bacterial DNA extraction, library preparation, whole-genome sequencing and bioinformatics﻿

GAS strains of *emm11* (*n*=11), *emm75* (*n*=17), *emm77* (*n*=22) and *emm92* (*n*=11) were grown on SBA and inspected for purity prior to DNA extraction. DNA was extracted using the Qiagen DNeasy blood and tissue kit (Qiagen) with modifications for Gram-positive bacteria as we have previously described [11]. DNA quantity and quality were measured using a NanoDrop instrument and Qubit. Short-read sequencing libraries were prepared using the Illumina Nextera flex sequencing kit as per the manufacturer’s instructions and 300 bp paired-end sequence obtained using an Illumina MiSeq instrument. Long-read sequencing libraries were prepared using the Oxford Nanopore rapid barcoding kit as per the manufacturer’s instructions and sequence obtained using an Oxford Nanopore GridION instrument. Complete genome assembly was performed using both long- and short-read sequences for TSPY155 (*emm11*), TSPY208 (*emm75*), TSPY165 (*emm77*), TSPY453 (*emm77*) and TSPY556 (*emm92*). Details of bioinformatic methods used for genome assembly, polymorphism discovery and phylogenetics are provided in the Supplementary Material.

### Comparison to publicly available GAS genome sequences

Large collections of GAS genome sequences are available through the Sequence Read Archive (SRA) at the National Center for Biotechnology Information (NCBI). Specifically, beginning in 2015, the CDC Active Bacterial Core Surveillance (ABCs) programme began sequencing all GAS invasive isolates [12]. Currently, over 5000 invasive GAS isolates have been sequenced spanning the years 2015–2017 and are publicly available (BioProject accession number PRJNA395240). Available data include 1454 GAS strains from 2015 [12] and 3843 GAS strains from 2016 to 2017 (unpublished). Further, whole-genome sequences for over 500 GAS isolates were deposited by Public Health England (PHE) in 2014 as part of a study to investigate an increase in scarlet fever (BioProject accession number PRJEB13551) [13]. Together, the two datasets (ABCs and PHE) provide temporally matched and geographically diverse GAS strains for comparison to our study population in Houston, TX, USA. A summary of the available GAS strain sequences (including those sequenced in the current study) is provided in Tables S1–S3, (available with the online version of this article).

### Statistics

Invasive indices for individual *emm* types were calculated by dividing the frequency of occurrence in invasive disease by the frequency of occurrence in pharyngeal disease. A resistance ratio was calculated by dividing the observed frequency of resistance to any antimicrobial within a single *emm* type by the frequency of resistance in the reference population. For example, to calculate the resistance ratio for *emm12* GAS, we divided the observed rate of resistance (any antimicrobial) within *emm12* (5/145) by the expected rate of resistance within the total population (100/929). Comparisons in the frequency of antimicrobial resistance between disease types were performed using Fisher’s exact test. The Mann–Whitney U test was used to compare invasive indices and resistance ratios between high- and low-frequency resistant groups. All calculations were performed in GraphPad/Prism v 8. *P* values less than 0.05 were considered significant for all statistical tests.

### Sequence data availability

All short- and long-read sequencing data has been deposited at the NCBI under BioProject accession number PRJNA494557. Complete genome sequence is available at the NCBI for TSPY155 (CP032699), TSPY165 (CP033336), TSPY208 (CP033335), TSPY453 (CP033337) and TSPY556 (CP032700 and CP032701).

## Results

### High rates of antimicrobial resistance associated with particular *emm* and disease types

We identified a total of 100 (10.8 %) GAS strains with resistance to at least one antimicrobial. A summary of the observed resistance phenotypes is given in Table 1. All GAS strains showed susceptibility to penicillin (data not shown). Tetracycline resistance was observed in 52 GAS strains (5.6 %). Using a combination of disc diffusion and MICs, we categorized resistance to erythromycin and clindamycin as M (macrolide resistance only), cMLS (constitutive resistance to macrolides and lincosamides) or iMLS (resistance to macrolide and inducible resistance to clindamycin) phenotypes. A total of 65 (7.0 %) GAS strains were identified with M/MLS (macrolide-lincosamide-streptogramin) phenotypes with iMLS accounting for the majority (39/65, 60 %) (Table 1).

**Table 1.** Frequency of antimicrobial resistance by GAS disease classification

Resistance phenotype	INV (%) [*n*=263]	SSTI (%) [*n*=235]	PHG (%) [*n*=431]	Total (%) [*n*=929]
M/MLS M cMLS iMLS	26 (9.9)* 6 (2.3) 8 (3.0) 12 (4.6)	22 (9.4) – 4 (1.7) 18 (7.7)	17 (3.9)† 6 (1.4) 2 (0.5) 9 (2.1)	65 (7.0) 12 (1.3) 14 (1.5) 39 (4.2)
TE	15 (5.7)	20 (8.3)*	17 (3.9)†	52 (5.6)
**TOTAL‡**	33 (12.5)*	34 (14.5)*	33 (7.7)†	100 (10.8)

*P<0.05 (Fisher’s exact test) compared to pharyngitis.

†*P*<0.05 (Fisher’s exact test) compared to combined non-pharyngeal (invasive+SSTI).

‡Total includes 6 GAS isolates with resistance to chloramphenicol (*n*=5) and levofloxacin (*n*=1).

cMLS, Constitutive MLS; iMLS, inducible MLS; INV, invasive; M, macrolide; MLS, macrolide-lincosamide-streptogramin; PHG, pharyngeal; TE, tetracycline.

**Fig. 1.**
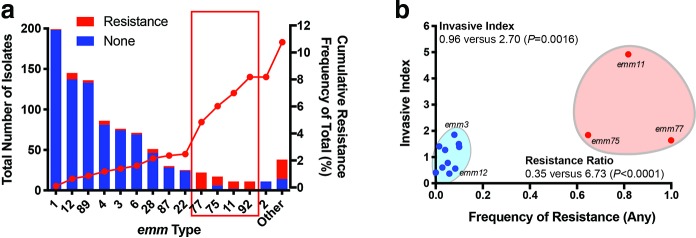
Frequency of antibiotic resistance by *emm* type and association with invasive disease in GAS strains from Houston, TX, USA. (a) Total number of isolates by *emm* type (left *y*-axis) and frequency of any antibiotic resistance (red bars). Only those *emm* types with at least 10 observations are indicated. Cumulative frequency of resistance is indicated by the red line (right *y*-axis). (b) Invasive index versus frequency of antibiotic resistance by *emm* type in the Houston GAS strains. Cumulative invasive indices and resistance ratios (see Methods and Table S1) are indicated between low-frequency (blue shaded circle) and high-frequency (red shaded circle) resistant *emm* types (Fisher’s exact test). *emm92* GAS strains are not shown as no pharyngeal isolates were identified in the Houston GAS collection for calculation of the invasive index.

We discovered unique relationships when examining the frequency of any antimicrobial resistance within *emm* types. Among *emm* types with at least 10 observations, we found that *emm* types 11, 75, 77 and 92 had high-frequency (e.g. >50 %) within-*emm* type resistance (Fig. 1a, Table S4). Collectively, the four *emm* types showed >85 % resistance to at least one antimicrobial but constituted<7 % of the GAS strain population – an eightfold greater frequency (i.e. resistance ratio, see Methods) than expected compared to all other *emm* types (*P*=0.003). The observed significance was not due to a single resistance phenotype among the high-resistance *emm* types but, rather, to both tetracycline (1.1 versus 72.1 %, *P*<0.0001) and M/MLS (2.1 versus 49.2 %, *P*<0.0001) resistance phenotypes (Table S4).

Using GAS disease type definitions established in our original molecular epidemiology study [9], we compared the frequency of resistance phenotypes between GAS strains derived from invasive infection, SSTI and pharyngeal infection. We observed a significantly higher frequency of any resistance phenotype in non-pharyngeal (i.e. invasive and SSTI) strains (67/498, 13.5 %; Table 1) compared to pharyngeal-derived GAS (33/431, 7.7 %, *P*=0.0042). The significantly higher frequency of resistance in non-pharyngeal GAS strains was retained when comparing tetracycline resistance (7.0 versus 3.9 %, *P*=0.045) and for M/MLS (9.6 versus 3.9 %, *P*=0.0007). Moreover, GAS *emm* types 11, 75, 77 and 92 accounted for the majority of strains with any resistance phenotype in the invasive group (24/33, 72.7 %) (Tables 1 and Table S4). Given that *emm* types 11, 75, 77 and 92 showed high within-*emm* type resistance rates and accounted for the majority of MLS resistance among invasive strains, we next postulated that the high-frequency resistant *emm* types were more prevalent in invasive compared to pharyngeal disease. The GAS invasive index is defined as the frequency of *emm* type occurrence in invasive disease divided by the frequency of the same *emm* type in pharyngeal disease derived from the same population over a similar time period and is considered an indicator of the invasive potential of a GAS *emm* type. Surprisingly, we observed a direct relationship between the frequency of any antimicrobial resistance and invasive index (Fig. 1b). Additionally, we calculated cumulative invasive indices and resistance ratios (see Methods) and compared high- and low-frequency resistant *emm* types. We observed a significantly greater invasive index (2.70 versus 0.96, *P*=0.03) and resistance ratio (8.07 versus 0.26, *P*=0.003) in high-frequency compared to low-frequency resistant *emm* types, respectively (Table S4, Fig. 1b). These data suggest overrepresentation of high-frequency resistance strains in invasive disease compared to low-resistance *emm* types.

### Whole-genome sequencing of high-frequency resistant GAS *emm* types reveals distinct differences and similarities in mobile genetic element content

We sought to better understand the genes and mobile genetic elements associated with antimicrobial resistance in *emm* types with high-frequency resistance (*emm11*, *emm75*, *emm77* and *emm92*). Using both long- and short-read genome sequences (see Methods), we sequenced to completion representative genomes of each high-frequency resistant *emm* type (Table S5). Whole-genome sequence (short-read only) was obtained from the remaining *emm11* (*n*=10), *emm75* (*n*=16), *emm77* (*n*=21) and *emm92* (*n*=10) GAS isolates for comparison to their respective completed, reference genome. In addition to our local GAS strain collection, we compared genome sequences from temporally matched GAS *emm* types from the CDC ABCs [12] and PHE, UK [13] (see Tables S1–S3 and Methods).

### *e* *mm11* GAS strains are defined by the presence of Tn*916* and Tn*916*-like elements

**Fig. 2.**
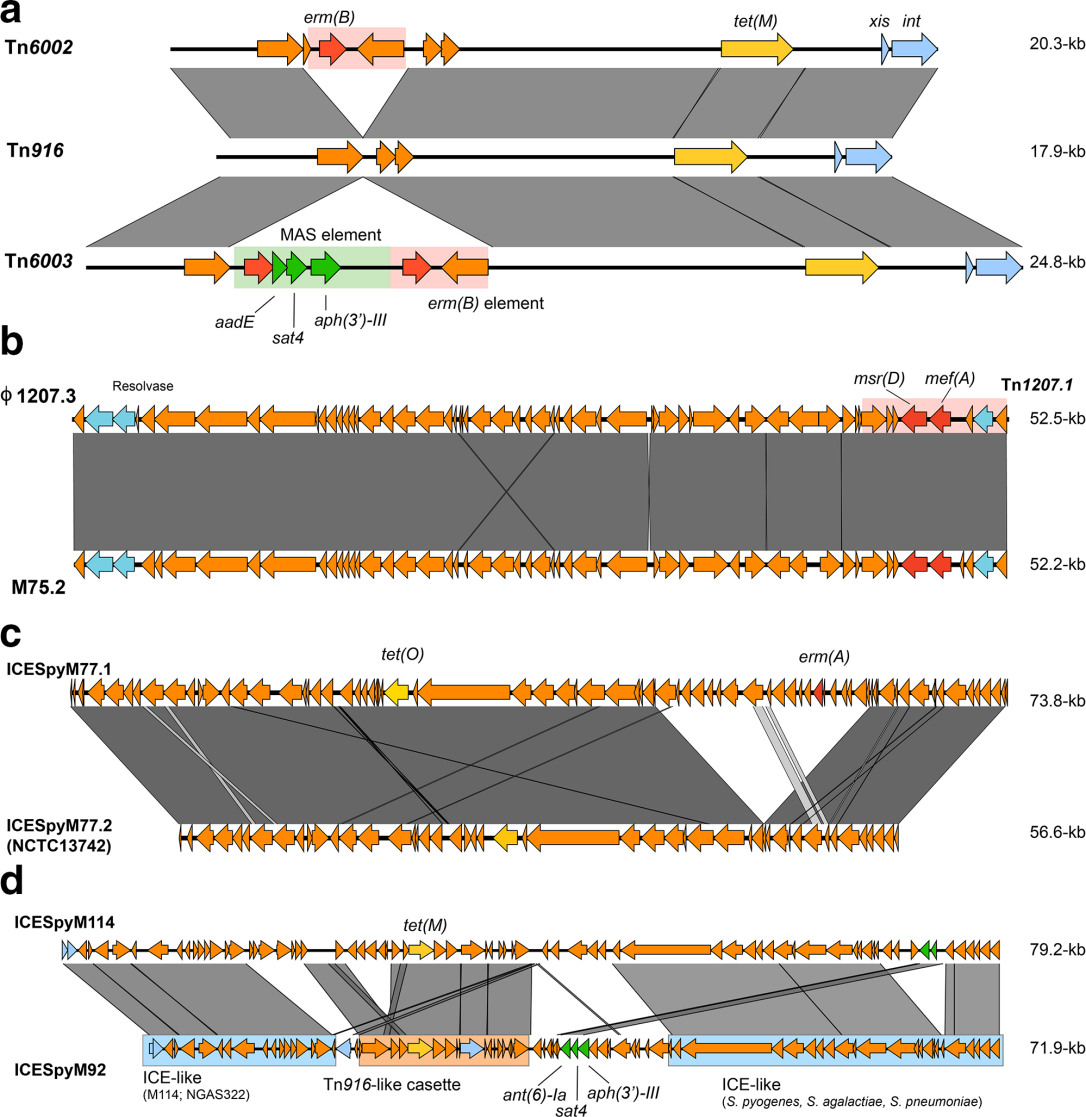
blast comparisons of mobile elements harbouring antibiotic-resistance genes identified among *emm11*, *emm75*, *emm77* and *emm92* GAS in Houston, TX. (a) Tn*916* and Tn*916-*like (Tn*6002* and Tn*6003*) elements identified in *emm11*, *emm75* and *emm77* GAS strains. Tn*6003* contains two copies of *erm(B)* (red) that may facilitate excision of the macrolide-aminoglycoside-streptothricin (MAS) element in formation of Tn*6002* [14]. (b) M75.2 prophage containing Tn*1207.1* (red shaded box) compared to the previously described ϕ1207.3 [15]. (c) ICE identified in *emm77* GAS strains. ICESpyM77.2 is identical to that in NCTC13742 (NCBI accession number LS483386) and differs from ICESpyM77.1 by the absence of a 16.6 kb segment containing *erm(A)* (red). (d) ICE identified in *emm92* (ICESpyM92) compared to ICE in NGAS322 (ICESpyM114) [18]. Shaded regions represent >95 % nucleotide identity in all comparisons.

Tetracycline resistance, *tet(M)*, was most common among *emm11* GAS strains in Houston, TX, occurring in 82 % (Table 2) of isolates. Erythromycin and clindamycin resistance conferred by *erm(B)* was present in 7/11 (63.6 %) isolates. In contrast, a cluster of 21 strains from the CDC collection contained the *erm(A)* gene, but did not include any strains from Houston (Fig. S1). GAS *emm11* strains were not identified in the 2014 PHE, UK collection. Three Houston isolates contained a gene for aminoglycoside resistance [*aph(3')-III*] resulting in high-level resistance to kanamycin, but susceptibility to streptomycin and gentamicin (Table S6). All resistance genes were found on Tn*916* or the closely related Tn*6002* or Tn*6003* (Fig. 2a, Table 2). Comparing our isolates to the geographically diverse GAS collection from the CDC, we found high conservation of Tn*6002* and Tn*6003* mobile elements (Table 2, Fig. S1). It has been previously noted that spontaneous excision of the macrolide-aminoglycoside-streptothricin (MAS) element from Tn*6003* may result in the formation of Tn*6002* (Fig. 2a), potentially contributing to the variable aminoglycoside resistance identified in both the Houston and CDC collections [14].

**Table 2.** Summary of antibiotic-resistance gene frequency in the Houston collection compared to the CDC/PHE, UK sets

*emm*	MGE	Resistance genes*	Strain count (%)	
			Houston	CDC/UK
11	Tn*916* Tn*6002* Tn*6003*	*tet(M)* *tet(M), erm(B)* *tet(M), erm(B), aph(3')-III*	2 (18.2) 4 (36.4) 3 (27.3)	2 (1.0) 58 (29.1) 79 (39.7)
75	M75.2/ϕ1207.3† Tn*916*	*mef(A), msr(D)* *tet(M)*	8 (42.1) 2 (10.5)	12 (12.7) 9 (9.6)‡
77	Tn*916* ICESpyM77.1 ICESpyM77.2	*tet(M)* *tet(O)* *tet(O), erm(A)*	3 (13.6) 12 (54.5) 7 (31.8)	28 (14.4) 57 (29.2) 72 (36.9)§
92	ICESpyM92 pRW35||	*tet(M), ant* *(6)* *-Ia, aph(3′)-III* *erm(T)*	11 (100) 11 (100)	233 (98.3) 228 (96.2)

*Resistance genes defined by the presence in the Houston, TX, collection.

†High identity (>99 %) of M75.2 to ϕ1207.3 [15].

‡No UK *emm75* GAS strains contained resistance elements. All nine CDC GAS isolates possessed Tn*6002*.

§Does not include 23 CDC GAS strains with *erm(T)*/*tet(M)* genotype.

||Presence of ICESpyM92 and plasmid pRW35 [17] not mutually exclusive.

mobile genetic element (MGE); Centers for Disease Control and Prevention (CDC); United Kingdom (UK)

### Macrolide resistance in Houston *emm75* GAS is attributable to prophage 1207.3

Resistance to erythromycin in *emm75* GAS from Houston was attributable to the presence of *mef(A)/msr(D)* in eight (47.1 %) strains (Table 2). Whole-genome sequencing and phylogenetic analysis of *emm75* GAS from Houston (*n*=17), CDC (*n*=88) and PHE, UK (*n*=17) collections revealed two clusters, consisting of distinct multilocus sequence types (STs), and each with variable resistance phenotypes/genotypes (Fig. S2). GAS strains from Houston were found in both clusters, but were most frequent in the cluster with macrolide resistance due to *mef(A)/msr(D)* carried on Tn*1207.1* that is embedded within a prophage. The prophage (M75.2) is identical to that previously described conferring macrolide resistance in GAS (ϕ1207.3; Fig. 2b) [15]. Distinctly, UK *emm75* GAS isolates clustered together and lacked any identifiable resistance genes. A small cluster (*n*=9) of CDC *emm75* strains contained *erm(B)* and *tet(M)*, presumably due to Tn*6002*.

### ICEs are the predominant resistance-carrying mobile elements among *emm77*

The most common mobile genetic elements identified were ICEs among *emm77* and *emm92* GAS. Phylogenetic analysis of the *emm77* GAS isolates across all compared collections showed three clusters with distinct STs (Fig. S3). The primary cluster (ST63) was defined by the presence of a *tet(O)*- or *tet(O)/erm(A)*-containing ICEs (ICESpyM77.1 and ICESpyM77.2, respectively) and included the majority (19/22) of the Houston *emm77* GAS strains (Figs 2c and S3, Table 2). The remaining clusters consisted of two separate and distinct multilocus STs (ST133 and ST399) defined by the presence of Tn*916*-like elements differing from the ICE-containing *emm77* (ST63). Interestingly, a single Houston *erm(A)*-positive *emm77* GAS strain (TSPY453) showed constitutive resistance (cMLS) to clindamycin (Table S7). Mutations in the nucleotide sequence encoding the leader peptide immediately upstream of *erm(A)* are known to alter resistance phenotypes in GAS and other organisms [16]. We compared the upstream sequences of *erm(A)* in the seven Houston *emm77* strains and discovered two single nucleotide changes in TSPY453 that may alter *erm(A)* transcription, yielding the observed phenotype (Fig. S4).

### *e* *mm92* GAS strains appear to be highly clonal in the USA suggesting recent emergence

The Houston *emm92* GAS strains displayed uniform resistance phenotypes and represent one of the only GAS *emm* types to be consistently associated with a plasmid [17]. The ICE identified in *emm92* (Fig. 2d) carried tetracycline resistance [*tet(M)*] and showed highest similarity to an ICE in NGAS322 (*emm114*) [18]. However, in contrast to the NGAS322 ICE, the ICE identified in *emm92* carried genes predicted to impart high-level aminoglycoside resistance [*ant(6)-Ia* and *aph(3′)-III*]. We performed MIC determinations to several aminoglycosides and confirmed high-level resistance in the Houston *emm92* GAS strains (Table S6). Resistance to erythromycin and inducible resistance to clindamycin (iMLS phenotype) is the result of *erm(T)* carried on a previously described plasmid (pRW35) [17]. Importantly, the ICE conferring tetracycline and aminoglycoside resistance and the plasmid conferring the iMLS resistance phenotype appear to be highly conserved among *emm92* strains in the USA (Table 2).

**Fig. 3.**
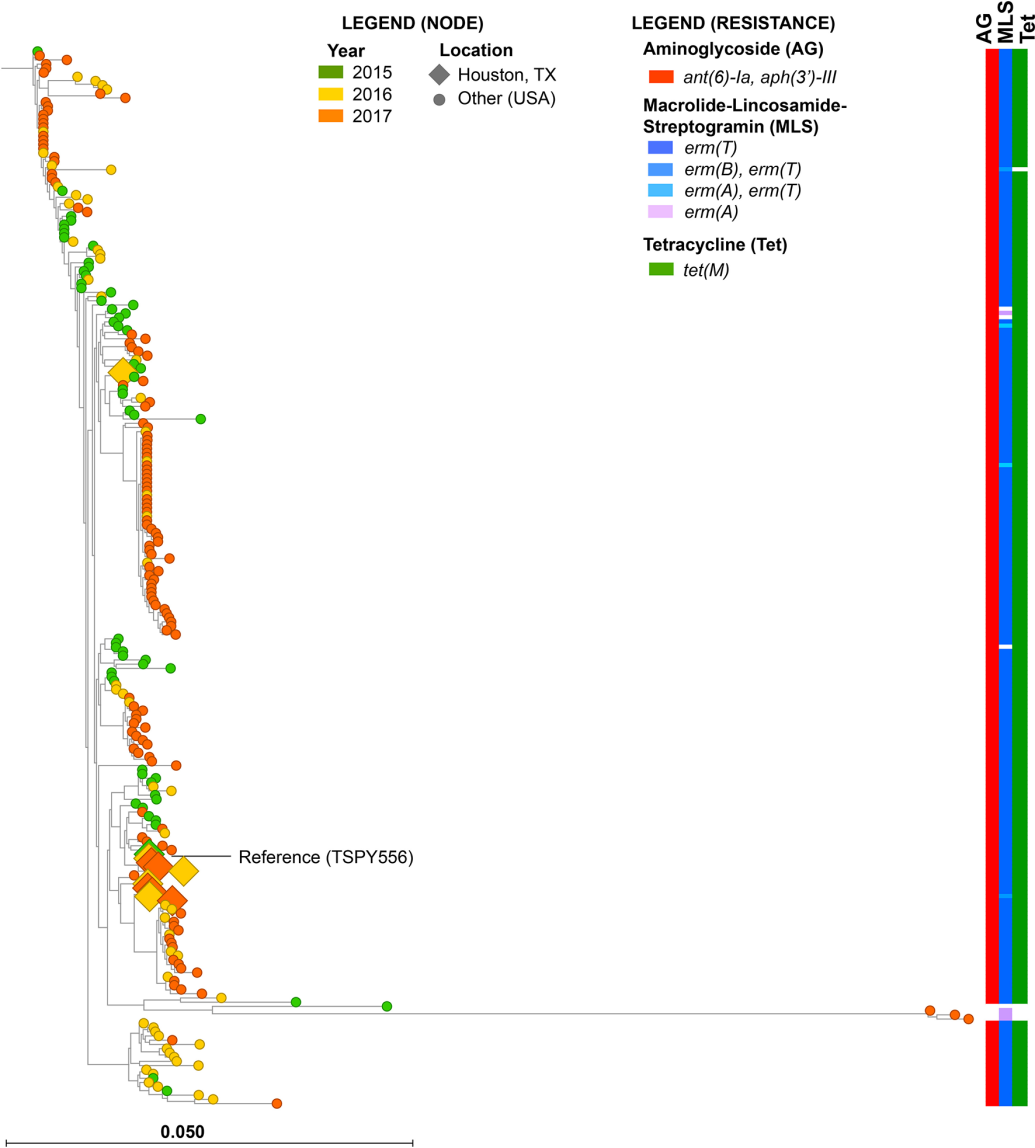
Phylogenetic reconstruction using 827 core biallelic SNP loci of *emm92* GAS strains from Houston (*n*=19) and the CDC ABCs (*n*=239) relative to the reference TSPY556. Node colour and shape indicate year of isolation and geographical location, respectively (see node legend). Resistance genotypes for aminoglycosides (AG), MLS and tetracycline (Tet) are shown on right, and coloured by allele (see resistance legend).

Whole-genome sequencing and phylogenetic analysis of Houston and CDC ABCs *emm92* GAS strains (none were identified in the 2014 UK study) [13, 12] revealed a highly clonal population belonging to the same ST that differed by a mean of only 20 SNPs, including several clusters of phylogenetically indistinguishable isolates from the reference (TSPY556) (Fig. 3). The majority (10/11) of the Houston *emm92* GAS isolates cluster very closely to the reference, differing by a mean of only 10 SNPs (range 3–17 SNPs) from TSPY556. Apart from a cluster of three isolates (Fig. 3), all *emm92* GAS strains contained identical mobile elements (e.g. ICE, plasmid and prophage).

## Discussion

Our study is one of the first to show a relationship between antimicrobial resistance and disease phenotype in GAS. Resistance to second-line antimicrobials has been identified as an emerging threat in the treatment of the diverse infections caused by GAS [6]. Multiple studies have described resistance patterns within single GAS *emm* types or clusters of infections, but few have examined associations between antimicrobial resistance and GAS disease types. Our data indicate that a small number of *emm* types disproportionately contribute to the overall antimicrobial resistance in our Houston GAS strain population. In addition, we show that *emm* types with high-frequency resistance are overrepresented among invasive compared to pharyngeal strains. Our findings were made possible through comprehensive disease surveillance (invasive, SSTI and pharyngeal), and emphasizes the need for continued and expanded surveillance in our and other communities. While the described *emm* types may not be highly prevalent, the high rates of antimicrobial resistance associated with the potential for enhanced virulence should cause concern among clinicians.

The rate of resistance to any antimicrobial (10.8 %) we observed is less than that in larger, nationwide studies conducted by the CDC ABCs (22.6 %) [12]. Higher antimicrobial-resistance frequencies have been reported, but tend to be limited to single *emm* types within a given community or locale. We observed macrolide resistance in 7 % of our GAS isolates. A large, multi-centre study examining pharyngeal GAS strains over a 3 year period (2000–2003) showed modest (4.1 %) resistance to macrolides [19] – similar to the rate of resistance observed in Houston pharyngeal strains (3.9 %). In 2015, nearly 14 % of the CDC ABCs invasive collection showed macrolide resistance [12], which may reflect differences in disease (invasive versus pharyngeal), but the two studies were performed on different populations and at different points in time. Examination of GAS macrolide resistance in the USA between 2002 and 2003 showed 6.8 % resistance [20], and included pharyngeal and non-pharyngeal sources as in our study. However, over 85 % of the isolates were defined as ‘throat’ and the study was unable to identify significant differences in the rate of macrolide resistance between pharyngeal and non-pharyngeal sources [20].

Our study does not provide direct evidence of enhanced virulence, but suggests an association between high-frequency resistance *emm* types and invasive disease. The effect on virulence of acquired antimicrobial resistance in bacteria is an area of profound interest in infectious diseases. Perhaps the best described associations between virulence and antimicrobial resistance are related to the acquisition of the staphylococcal cassette chromosome (SCC*mec*) that confers resistance to β-lactam antimicrobials in meticillin-resistant *Staphylococcus aureus* (MRSA). Community-acquired MRSA (CA-MRSA) strains demonstrate high virulence in animal models and tend to carry SCC*mec* types IV or V [21]. In contrast, the presence of a larger SCC*mec* type in hospital-acquired MRSA (HA-MRSA) strains results in reduced virulence in mice [22], but is likely dependent upon strain background [21]. Importantly, the presence of SCC*mec* is critical to determining appropriate antimicrobial therapy. Likewise, increased prevalence of MLS resistance phenotypes in GAS *emm* types associated with invasive disease may alter recommended adjunctive therapy with clindamycin for severe invasive disease (e.g. toxic shock syndrome).

Among the high-frequency resistant GAS *emm* types described in the Houston collection, we observed a disproportionate number in invasive disease compared to pharyngeal. Moreover, *emm11* and *emm92* GAS isolates demonstrate high-frequency resistance to multiple antimicrobials including macrolides, tetracyclines and aminoglycosides. We confirmed high-frequency resistance in *emm11* and *emm92* GAS strains by comparing to the geographically disparate strains captured by the CDC ABCs. Most striking was that *emm92* GAS strains collectively showed >98 % resistance in Houston and the CDC ABCs. Phylogenetic analysis of 250 *emm92* GAS strains showed a very high degree of relatedness and suggests a recent emergence, although *emm92* GAS strains have been described as early as 1992 in New Zealand [23] and 1997 in the USA [24]. Inasmuch as tetracycline resistance has been postulated to contribute to the emergence of pathogenic strains of group B *Streptococcus* [25, 26], it is tempting to speculate that the presence of the antimicrobial-resistance-containing elements in *emm11* and *emm92* GAS strains may contribute to their disease potential.

The high invasive indices of resistant *emm* types raises the question of how these strains are transmitted within the human population. Examination of our original study [9] showed that all *emm11* strains were derived from non-pharyngeal abscesses (e.g. lymph node), with the exception of two cases of pharyngeal space abscesses. Likewise, *emm92* strains originated from abscess (lymph node) or cellulitis, with only two cases derived from pharyngeal space abscesses. Our findings are reminiscent of the Canadian *emm59* epidemic that resulted in increased invasive disease, but was an unusual cause of pharyngitis [27]. It is possible that spread/transmission of *emm11* and *emm92* GAS strains occurs via direct skin-to-skin contact, as suggested for *emm59*. However, it is also possible that they are transmitted asymptomatically through pharyngeal spread. Moreover, as has been suggested for *erm(A)*-carrying *emm94* GAS [28] (pattern E), it is possible that the high frequency of resistance observed is due to increased transmissibility compared to susceptible strains of the same *emm* type. Currently, our dataset does not include carriage surveillance and further research is needed to directly address such scenarios.

Fundamental differences in GAS strain biology apart from invasiveness may account for differences in resistance frequency between *emm* types. GAS serotypes can be grouped based on genetic markers associated with *emm* genes and tissue tropism and include pharyngeal (pattern A–C), skin (pattern D) and generalists or no specificity (pattern E) strains [29]. It is intriguing that all highly resistant *emm* types (11, 75, 77 and 92) in our study belong to pattern E. It is possible that gene content unique to pattern E GAS strains (e.g. serum opacity factor, SOF) contributes to the higher frequency of antimicrobial resistance including a more ready acquisition of mobile genetic elements compared to pattern A–C strains. Additional investigation is needed into the association of *emm* pattern and antimicrobial resistance and any relationship into the emergence of resistance and potential contributions to virulence.

Our study has several limitations. We used disc diffusion to screen for antimicrobial resistance and performed MIC determinations (via Etest) on a subset of strains and antimicrobials. Thus, it is possible that MIC determinations (via broth microdilution or Etest) on all strains with all antimicrobials may identify more subtle phenotypes and alter our findings. However, we observed near 100 % resistance genotype–phenotype concordance in sequenced strains, indicating a high degree of accuracy in our screening method. We observed relatively small numbers of *emm11*, *emm75*, *emm77* and *emm92* GAS strains over a limited timespan (4 years), increasing the chance of type II error. We partially mitigated the effect of low numbers by including analyses of publicly available and temporally matched GAS strain sequences from the CDC ABCs collection and the UK. Finally, we sequenced only a subset of GAS *emm* types and it is undoubtedly likely that additional genetic elements associated with GAS antimicrobial resistance are circulating within our strain population.

Overall, our study provides important insight into relationships between GAS *emm* type, disease type and antimicrobial resistance. Our findings that specific *emm* types display high-frequency resistance and may disproportionately contribute to invasive disease warrant continued surveillance and deeper investigative studies into antimicrobial resistance–strain virulence relationships in GAS.

## Data bibliography

1. Chalker V, Jironkin A, Coelho J, Al-Shahib A, Platt S *et al*. Genome analysis following a national increase in Scarlet Fever in England 2014. BioProject number PRJEB13551.

2. Chochua S, Metcalf BJ, Li Z, Rivers J, Mathis S, *et al*. Population and Whole Genome Sequence Based Characterization of Invasive Group A Streptococci Recovered in the United States during 2015. BioProject accession# PRJNA395240.

## Supplementary Data

Supplementary File 1
