# Supplementary material for: Unexpected relationships between frequency of antimicrobial resistance, disease phenotype and emm type in group A Streptococcus
Source: Microb Genom. 2019 Nov 22;5(11):e000316. doi: 10.1099/mgen.0.000316 (PMC6927302; doi:10.1099/mgen.0.000316)
Supplement: Supplementary File 1 [file mgen-5-316-s001.pdf]

SUPPORTING INFORMATION

SUPPORTING METHODS

Genome assembly, polymorphism discovery, and bioinformatics. Long-read sequences were assembled using Canu v 1.7.1 (1) and resultant contiguous sequences (contigs) were imputed in the hybrid assembler Unicycler v 0.4.6 (2) that yielded a single complete chromosome for all strains. Complete genome sequences were submitted to the National Center for Biotechnology Information (NCBI) and annotated using NCBI's prokaryotic genome annotation pipeline (PGAP).

A custom in-house pipeline was used to identify polymorphisms (single nucleotide and insertions/deletions) relative to the *emm* type-matched reference including short-read sequences obtained from the sequence read archive (SRA) (3, 4). Short-read sequences were trimmed using Trimmomatic v 0.38 (5) and error correction performed using Musket v 1.1 (6). Corrected reads were subsequently mapped to the reference genome using SMALT v 0.7.6 (https://www.sanger.ac.uk/science/tools/smalt-0) and polymorphisms identified using freebayes v 1.2.0 (7). All polymorphisms were filtered for depth of coverage (≥15-fold coverage), polymorphism frequency (≥75%), and quality (phred score ≥10). Resultant variant call format (VCF) files were processed using the custom python scripts prephix and phrecon (https://github.com/codinghedgehog) and phylogenetic trees generated using FastTree v 2.1.10 (8). Phylogenetic trees were visualized and manipulated in CLC Genomics Workbench v 12.0.1 (Qiagen, Valencia, CA). De novo assembly of short-read sequences was performed using SPAdes (9) and resultant contigs used to search the

ResFinder database (Center for Genomic Epidemiology, https://cge.cbs.dtu.dk/services/ResFinder/) for antimicrobial resistance genes using the Microbial Genomics module (v 3.6.1) of the CLC Genomics Workbench. Nucleotide sequence comparisons and visualizations were made using Easyfig (10).

SUPPORTING TABLES

TABLE S1. Houston GAS strains used in whole genome sequencing.

Strain	Biosample[a]	*Emm*[b]	ST[c]	Year[d]	AG[e]	MLS	Tet	AG/MLS/Tet Phenotype[f]	Disease Type
TSPY155	SAMN10172638	11	403	2013		*erm(B)*	*tet(M)*	**S / R / R**	**Invasive**
TSPY376	SAMN10172639	11	403	2014		*erm(B)*	*tet(M)*	**S / R / R**	**Invasive**
TSPY555	SAMN10172640	11	403	2015		*erm(B)*	*tet(M)*	**S / R / R**	**Invasive**
TSPY682	SAMN10172641	11	403	2015				**S / S / S**	**Invasive**
TSPY711	SAMN10172642	11	403	2015	*aph(3')-III*	*erm(B)*	*tet(M)*	**R / R / R**	**Invasive**
TSPY784	SAMN10172643	11	403	2016			*tet(M)*	**S / S / R**	**SSTI**
TSPY942	SAMN10172644	11	403	2016		*erm(B)*	*tet(M)*	**S / R / R**	**SSTI**
TSPY1115	SAMN10172645	11	403	2016	*aph(3')-III*	*erm(B)*	*tet(M)*	**R / R / R**	**Invasive**
TSPY1165	SAMN10172646	11	403	2017	*aph(3')-III*	*erm(B)*	*tet(M)*	**R / R / R**	**Pharyngeal**
TSPY1172	SAMN10172647	11	403	2017			*tet(M)*	**S / S / R**	**SSTI**
TSPY1239	SAMN10172648	11	403	2017				**S / S / S**	**Pharyngeal**
TSPY197	SAMN10172650	75	49	2014				**- / S / S**	**Invasive**
TSPY208	SAMN10172651	75	49	2014		*mef(A), msr(D)*		**- / R / S**	**Pharyngeal**
TSPY282	SAMN10172652	75	39	2014				**- / R / S**	**Invasive**
TSPY392	SAMN10172653	75	49	2014		*mef(A), msr(D)*		**- / R / S**	**Invasive**
TSPY481	SAMN10172654	75	49	2014				**- / S / S**	**Pharyngeal**
TSPY519	SAMN10172655	75	NF	2014			*tet(M)*	**- / S / R**	**Invasive**
TSPY679	SAMN10172656	75	49	2015		*mef(A), msr(D)*		**- / R / S**	**Pharyngeal**
TSPY689	SAMN10172657	75	49	2015		*mef(A), msr(D)*		**- / R / S**	**Invasive**
TSPY690	SAMN10172658	75	49	2015				**- / S / S**	**Pharyngeal**
TSPY700	SAMN10172659	75	49	2015		*mef(A), msr(D)*	*tetA(46)*	**- / R / S**	**Pharyngeal**
TSPY748	SAMN10172660	75	49	2015		*mef(A), msr(D)*		**- / R / S**	**Pharyngeal**
TSPY923	SAMN10172661	75	150	2016				**- / S / S**	**Invasive**
TSPY979	SAMN10172662	75	49	2016		*mef(A), msr(D)*		**- / R / S**	**Invasive**
TSPY985	SAMN10172663	75	49	2016				**- / S / S**	**Invasive**
TSPY1075	SAMN10172664	75	49	2016				**- / S / S**	**Pharyngeal**
TSPY1078	SAMN10172665	75	NF	2016			*tet(M)*	**- / S / R**	**SSTI**
TSPY1091	SAMN10172666	75	49	2016		*mef(A), msr(D)*		**- / R / S**	**Invasive**
TSPY1122	SAMN10172667	75	49	2016				**- / S / S**	**SSTI**
TSPY1231	SAMN10172668	75	52	2017				**- / S / S**	**Pharyngeal**
TSPY165	SAMN10172669	77	133	2013			*tet(M)*	**- / S / R**	**Invasive**
TSPY221	SAMN10172670	77	399	2014			*tet(M)*	**- / S / R**	**Invasive**
TSPY311	SAMN10172671	77	63	2014		*erm(A)*	*tet(O)*	**- / R / R**	**Pharyngeal**
TSPY373	SAMN10172672	77	63	2014			*tet(O)*	**- / S / R**	**Pharyngeal**
TSPY377	SAMN10172673	77	63	2014		*erm(A)*	*tet(O)*	**- / R / R**	**SSTI**
TSPY453	SAMN10172674	77	63	2014		*erm(A)*	*tet(O)*	**- / R / R**	**SSTI**
TSPY477	SAMN10172675	77	63	2014			*tet(O)*	**- / S / R**	**SSTI**
TSPY482	SAMN10172676	77	63	2014			*tet(O)*	**- / S / R**	**Invasive**
TSPY656	SAMN10172677	77	63	2015			*tet(O)*	**- / S / R**	**Invasive**
TSPY658	SAMN10172678	77	63	2015		*erm(A)*	*tet(O)*	**- / R / R**	**Pharyngeal**
TSPY670	SAMN10172679	77	63	2015			*tet(O)*	**- / S / R**	**Invasive**
TSPY673	SAMN10172680	77	63	2015			*tet(O)*	**- / S / R**	**Pharyngeal**
TSPY695	SAMN10172681	77	63	2015			*tet(O)*	**- / S / R**	**SSTI**
TSPY788	SAMN10172682	77	63	2016			*tet(M)*	**- / S / R**	**Pharyngeal**
TSPY886	SAMN10172683	77	63	2016		*erm(A)*	*tet(O)*	**- / R / R**	**SSTI**
TSPY981	SAMN10172684	77	63	2016			*tet(O)*	**- / S / R**	**SSTI**
TSPY1030	SAMN10172685	77	63	2016			*tet(O)*	**- / S / R**	**Invasive**
TSPY1126	SAMN10172686	77	63	2016		*erm(A)*	*tet(O)*	**- / R / R**	**Invasive**
TSPY1151	SAMN10172687	77	63	2017			*tet(O)*	**- / S / R**	**Pharyngeal**
TSPY1153	SAMN10172688	77	63	2017		*erm(A)*	*tet(O)*	**- / R / R**	**SSTI**
TSPY1177	SAMN10172689	77	63	2017			*tet(O)*	**- / S / R**	**SSTI**
TSPY1260	SAMN10172690	77	63	2017			*tet(O)*	**- / S / R**	**Pharyngeal**
TSPY556	SAMN10172691	92	82	2015	*ant(6)-Ia, aph(3')-III*	*erm(T)*	*tet(M)*	**R / R / R[g]**	**Invasive**
TSPY783	SAMN10172692	92	82	2016	*ant(6)-Ia, aph(3')-III*	*erm(T)*	*tet(M)*	**R / R / R**	**Invasive**
TSPY854	SAMN10172693	92	82	2016	*ant(6)-Ia, aph(3')-III*	*erm(T)*	*tet(M)*	**R / R / R**	**Invasive**

TSPY1042	SAMN10172694	92	82	2016	*ant(6)-Ia, aph(3')-III*	*erm(T)*	*tet(M)*	**R / R / R**	**SSTI**
TSPY1076	SAMN10172695	92	82	2016	*ant(6)-Ia, aph(3')-III*	*erm(T)*	*tet(M)*	**R / R / R**	**Invasive**
TSPY1089	SAMN10172696	92	82	2016	*ant(6)-Ia, aph(3')-III*	*erm(T)*	*tet(M)*	**R / R / R**	**SSTI**
TSPY1195	SAMN10172697	92	82	2017	*ant(6)-Ia, aph(3')-III*	*erm(T)*	*tet(M)*	**R / R / R**	**Invasive**
TSPY1218	SAMN10172698	92	82	2017	*ant(6)-Ia, aph(3')-III*	*erm(T)*	*tet(M)*	**R / R / R**	**SSTI**
TSPY1227	SAMN10172699	92	82	2017	*ant(6)-Ia, aph(3')-III*	*erm(T)*	*tet(M)*	**R / R / R**	**Invasive**
TSPY1285	SAMN10172700	92	82	2017	*ant(6)-Ia, aph(3')-III*	*erm(T)*	*tet(M)*	**R / R / R**	**SSTI**
TSPY1287	SAMN10172701	92	82	2017	*ant(6)-Ia, aph(3')-III*	*erm(T)*	*tet(M)*	**R / R / R**	**SSTI**

[a] Biosample number under BioProject PRJNA494557.

[b] GAS *emm* type determined as described in Methods.

[c] Sequence type (ST) determined as described in Methods.

[d] Year of isolation.

[e] Antimicrobial resistance genes identified through bioinformatics pipeline defined in Methods for aminoglycosides (AG), macrolide-lincosamide-streptogramin (MLS), tetracycline (TET).

[f] Antimicrobial resistance phenotypes of susceptible (S), intermediate (I), and resistant (R) as determined by Etest or by broth microdilution in the case of aminoglycoside resistance in *emm11* and *emm92* isolates. A dash (-) indicates phenotype not tested.

[g] Tetracycline susceptibility in all *emm92* GAS showed resistance by disk diffusion but intermediate by Etest.

TABLE S2. CDC ABCs strains used in this study.

Biosample[a]	*Emm*[b]	ST[c]	Year[d]	AG[e]	MLS	Tet
SAMN07154200	11	403	2015	*aph(3')-III*	*erm(B)*	*tet(M)*
SAMN07154022	11	403	2015			
SAMN07154013	11	403	2015	*aph(3')-III*	*erm(B)*	*tet(M)*
SAMN07154148	11	403	2015		*erm(B)*	*tet(M)*
SAMN07154044	11	403	2015			
SAMN07154047	11	403	2015		*erm(B)*	*tet(M)*
SAMN07154333	11	403	2015			
SAMN07154365	11	403	2015	*aph(3')-III*	*erm(B)*	*tet(M)*
SAMN07154415	11	403	2015			
SAMN07154002	11	403	2015		*erm(A)*	
SAMN07154174	11	403	2015	*aph(3')-III*	*erm(B)*	*tet(M)*
SAMN07154035	11	403	2015	*aph(3')-III*	*erm(A)*	*tet(M)*
SAMN07154375	11	403	2015	*aph(3')-III*	*erm(B)*	*tet(M)*
SAMN07154249	11	403	2015		*erm(B)*	*tet(M)*
SAMN07154100	11	403	2015		*erm(B)*	*tet(M)*
SAMN07154134	11	403	2015			
SAMN07153831	11	403	2015		*erm(B)*	*tet(M)*
SAMN07153877	11	403	2015	*aph(3')-III*	*erm(B)*	*tet(M)*
SAMN07153542	11	403	2015		*erm(A)*	*tet(M)*
SAMN07153543	11	403	2015		*erm(B)*	*tet(M)*
SAMN07153661	11	403	2015			
SAMN07153563	11	403	2015			
SAMN07153811	11	403	2015	*aph(3')-III*	*erm(B)*	*tet(M)*
SAMN07153939	11	403	2015	*aph(3')-III*	*erm(B)*	*tet(M)*
SAMN07153523	11	403	2015		*erm(B)*	*tet(M)*
SAMN07153636	11	403	2015	*aph(3')-III*	*erm(B)*	*tet(M)*
SAMN07153839	11	403	2015		*erm(B)*	*tet(M)*
SAMN07153841	11	403	2015	*aph(3')-III*	*erm(B)*	*tet(M)*
SAMN07153871	11	403	2015		*erm(B)*	*tet(M)*
SAMN07153834	11	403	2015	*aph(3')-III*	*erm(B),isa(C)*	*tet(M)*
SAMN07153531	11	403	2015		*erm(B)*	*tet(M)*
SAMN07153530	11	403	2015		*erm(B)*	*tet(M)*
SAMN07153587	11	403	2015	*aph(3')-III*	*erm(B)*	*tet(M)*
SAMN07153851	11	403	2015		*erm(B)*	*tet(M)*
SAMN07153956	11	403	2015			
SAMN07153276	11	403	2015	*aph(3')-III*	*erm(B)*	*tet(M)*
SAMN07153371	11	403	2015	*ant(6)-Ia*	*erm(A)*	*tet(M)*
SAMN07152980	11	403	2015			
SAMN07153216	11	403	2015		*erm(B)*	*tet(M)*
SAMN07153339	11	403	2015	*aph(3')-III*	*erm(B)*	*tet(M)*
SAMN07153083	11	403	2015		*erm(B)*	*tet(M)*
SAMN07153194	11	403	2015			
SAMN07153301	11	403	2015			
SAMN07153348	11	403	2015	*aph(3')-III*	*erm(B)*	*tet(M)*
SAMN07153312	11	403	2015			
SAMN07153157	11	403	2015	*aph(3')-III*	*erm(B)*	*tet(M)*
SAMN07153195	11	907	2015			*tet(M)*
SAMN07153029	11	403	2015	*aph(3')-III*	*erm(B)*	*tet(M)*
SAMN07153324	11	403	2015		*erm(B)*	*tet(M)*
SAMN07153323	11	403	2015		*erm(B)*	*tet(M)*
SAMN07153315	11	403	2015			
SAMN08691522	11	403	2016			
SAMN08690626	11	403	2016	*aph(3')-III*	*erm(B)*	*tet(M)*
SAMN08691305	11	403	2016		*erm(B)*	*tet(M)*
SAMN08691063	11	403	2016			
SAMN08691462	11	403	2016	*aph(3')-III*	*erm(B)*	*tet(M)*
SAMN08691471	11	403	2016		*erm(B)*	*tet(M)*
SAMN08691010	11	403	2016		*erm(B)*	*tet(M)*
SAMN08691383	11	403	2016	*aph(3')-III*	*erm(B)*	*tet(M)*
SAMN08691169	11	403	2016		*erm(B)*	*tet(M)*
SAMN08690938	11	403	2016			
SAMN08691095	11	403	2016	*aph(3')-III*	*erm(B)*	*tet(M)*
SAMN08690789	11	403	2015	*aph(3')-III*	*erm(B)*	*tet(M)*

SAMN08690934	11	403	2016	*aph(3')-III*	*erm(B)*	*tet(M)*
SAMN08691400	11	403	2016	*aph(3')-III*	*erm(B)*	*tet(M)*
SAMN08691399	11	403	2016			
SAMN08690671	11	403	2016	*aph(3')-III*	*erm(B)*	*tet(M)*
SAMN08690674	11	403	2016	*aph(3')-III*	*erm(B)*	*tet(M)*
SAMN08691482	11	403	2016		*erm(B)*	*tet(M)*
SAMN08690636	11	403	2016	*aph(3')-III*	*erm(B)*	*tet(M)*
SAMN08691264	11	403	2016		*erm(B)*	*tet(M)*
SAMN08691257	11	403	2016		*erm(B)*	*tet(M)*
SAMN08690706	11	403	2016	*aph(3')-III*	*erm(B)*	*tet(M)*
SAMN08691214	11	403	2016	*aph(3')-III*	*erm(B)*	*tet(M)*
SAMN08690877	11	403	2016		*erm(B)*	*tet(M)*
SAMN08691355	11	403	2016	*aph(3')-III*	*erm(B)*	*tet(M)*
SAMN08690991	11	952	2016			
SAMN08691274	11	403	2016	*aph(3')-III*	*erm(B)*	*tet(M)*
SAMN08690713	11	403	2016	*aph(3')-III*	*erm(B)*	*tet(M)*
SAMN08691483	11	403	2016		*erm(B)*	*tet(M)*
SAMN08692115	11	403	2016		*erm(B)*	*tet(M)*
SAMN08692120	11	403	2017	*aph(3')-III*	*erm(B)*	*tet(M)*
SAMN08692123	11	403	2017	*aph(3')-III*	*erm(B)*	*tet(M)*
SAMN08691587	11	403	2016	*aph(3')-III*	*erm(B)*	*tet(M)*
SAMN08691874	11	403	2016		*erm(B)*	*tet(M)*
SAMN08692184	11	403	2017			
SAMN08692283	11	403	2017	*aph(3')-III*	*erm(B)*	*tet(M)*
SAMN08692461	11	403	2017		*erm(B)*	*tet(M)*
SAMN08691889	11	403	2016		*erm(B)*	*tet(M)*
SAMN08692271	11	403	2017		*erm(B)*	*tet(M)*
SAMN08692238	11	403	2017	*aph(3')-III*	*erm(B)*	*tet(M)*
SAMN08691826	11	403	2016		*erm(B)*	*tet(M)*
SAMN08691802	11	403	2016		*erm(B)*	*tet(M)*
SAMN08691699	11	403	2016	*aph(3')-III*	*erm(B)*	*tet(M)*
SAMN08692360	11	403	2016		*erm(B)*	*tet(M)*
SAMN08692218	11	403	2017	*aph(3')-III*	*erm(B)*	*tet(M)*
SAMN08692277	11	403	2017	*aph(3')-III*	*erm(B)*	*tet(M)*
SAMN08692282	11	403	2017	*aph(3')-III*	*erm(B)*	*tet(M)*
SAMN08691723	11	403	2016	*aph(3')-III*	*erm(B)*	*tet(M)*
SAMN08691861	11	403	2016		*erm(B)*	*tet(M)*
SAMN08692087	11	403	2016	*aph(3')-III*	*erm(B)*	*tet(M)*
SAMN08692018	11	403	2016	*aph(3')-III*	*erm(A)*	*tet(M)*
SAMN08692015	11	403	2016			
SAMN08692371	11	403	2016		*erm(B)*	*tet(M)*
SAMN08692017	11	403	2016	*aph(3')-III*	*erm(A)*	*tet(M)*
SAMN08691617	11	403	2016	*aph(3')-III*	*erm(B)*	*tet(M)*
SAMN08692010	11	403	2016			
SAMN08692210	11	403	2016		*erm(B)*	*tet(M)*
SAMN08692476	11	403	2017		*erm(B)*	*tet(M)*
SAMN08692137	11	403	2016		*erm(B)*	*tet(M)*
SAMN08691659	11	403	2016	*aph(3')-III*	*erm(B)*	*tet(M)*
SAMN08691871	11	403	2016	*aph(3')-III*	*erm(B)*	*tet(M)*
SAMN08692176	11	403	2017	*aph(3')-III*	*erm(B)*	*tet(M)*
SAMN08691777	11	403	2016	*aph(3')-III*	*erm(B)*	*tet(M)*
SAMN08692201	11	403	2016	*aph(3')-III*	*erm(B)*	*tet(M)*
SAMN08692125	11	403	2017	*aph(3')-III*	*erm(B)*	*tet(M)*
SAMN08691734	11	403	2016		*erm(B)*	*tet(M)*
SAMN08691880	11	403	2016		*erm(B)*	*tet(M)*
SAMN08691877	11	403	2016			
SAMN08691988	11	403	2016		*erm(B)*	*tet(M)*
SAMN08692073	11	403	2016		*erm(B)*	*tet(M)*
SAMN08692339	11	952	2016			
SAMN08691566	11	403	2016	*aph(3')-III*	*erm(B)*	*tet(M)*
SAMN08692090	11	403	2016	*aph(3')-III*	*erm(B)*	*tet(M)*
SAMN08692789	11	403	2017	*aph(3')-III*	*erm(B)*	*tet(M)*
SAMN08692828	11	403	2017			
SAMN08692702	11	1047	2017		*erm(B)*	*tet(M)*
SAMN08692547	11	403	2017			
SAMN08692698	11	403	2017	*aph(3')-III*	*erm(B)*	*tet(M)*

SAMN08692816	11	403	2017			
SAMN08692783	11	403	2017		*erm(B)*	*tet(M)*
SAMN08692624	11	403	2017		*erm(B)*	*tet(M)*
SAMN08692593	11	403	2017	*aph(3')-III*	*erm(B)*	*tet(M)*
SAMN08692716	11	403	2016	*aph(3')-III*	*erm(B)*	*tet(M)*
SAMN08692610	11	403	2017		*erm(B)*	*tet(M)*
SAMN08692644	11	403	2017	*aph(3')-III*	*erm(B)*	*tet(M)*
SAMN08692599	11	403	2017	*aph(3')-III*	*erm(B)*	*tet(M)*
SAMN08692962	11	403	2017		*erm(B)*	*tet(M)*
SAMN08692963	11	403	2017		*erm(B)*	*tet(M)*
SAMN08693151	11	403	2017	*aph(3')-III*	*erm(B)*	*tet(M)*
SAMN08693000	11	403	2017	*aph(3')-III*		*tet(M)*
SAMN08693053	11	403	2017	*aph(3')-III*	*erm(B)*	*tet(M)*
SAMN08692959	11	403	2017	*aph(3')-III*	*erm(B),isa(C)*	*tet(M)*
SAMN08692958	11	403	2017		*erm(B)*	*tet(M)*
SAMN08692860	11	403	2017		*erm(B)*	*tet(M)*
SAMN08693011	11	403	2017	*aph(3')-III*	*erm(B)*	*tet(M)*
SAMN08693256	11	403	2017		*erm(A)*	*tet(M)*
SAMN08693254	11	403	2017		*erm(A)*	*tet(M)*
SAMN08693255	11	403	2017		*erm(A)*	*tet(M)*
SAMN09849266	11	403	2017	*aph(3')-III*	*erm(B)*	*tet(M)*
SAMN09849748	11	403	2017		*erm(A)*	*tet(M)*
SAMN09849870	11	403	2017		*erm(A)*	*tet(M)*
SAMN09849872	11	403	2017		*erm(A)*	*tet(M)*
SAMN09849526	11	403	2017	*aph(3')-III*	*erm(B)*	*tet(M)*
SAMN09849531	11	403	2017	*aph(3')-III*	*erm(B)*	*tet(M)*
SAMN09849532	11	403	2017		*erm(B)*	*tet(M)*
SAMN09849649	11	403	2017	*aph(3')-III*	*erm(B)*	*tet(M)*
SAMN09849600	11	403	2017	*aph(3')-III*	*erm(B)*	*tet(M)*
SAMN09849236	11	403	2017	*aph(3')-III*	*erm(B)*	*tet(M)*
SAMN09849827	11	403	2017			
SAMN09849205	11	403	2017		*erm(B)*	*tet(M)*
SAMN09849613	11	403	2017	*aph(3')-III*	*erm(B)*	*tet(M)*
SAMN09849723	11	403	2018		*erm(A)*	*tet(M)*
SAMN09848982	11	403	2016		*erm(B)*	*tet(M)*
SAMN09848980	11	403	2016			
SAMN09849784	11	403	2017	*aph(3')-III*	*erm(B)*	*tet(M)*
SAMN09849882	11	403	2017			
SAMN09849879	11	403	2017		*erm(A)*	*tet(M)*
SAMN09849876	11	403	2017		*erm(A)*	*tet(M)*
SAMN09849341	11	403	2017	*aph(3')-III*	*erm(B)*	*tet(M)*
SAMN09849338	11	403	2017	*aph(3')-III*	*erm(B)*	*tet(M)*
SAMN09849837	11	403	2017		*erm(A)*	*tet(M)*
SAMN09849054	11	403	2017	*aph(3')-III*	*erm(B)*	*tet(M)*
SAMN09849469	11	403	2017	*aph(3')-III*	*erm(B)*	*tet(M)*
SAMN09849510	11	403	2017			
SAMN09849332	11	403	2017			
SAMN09848917	11	403	2016	*aph(3')-III*	*erm(B)*	*tet(M)*
SAMN09849239	11	403	2017	*aph(3')-III*	*erm(B)*	*tet(M)*
SAMN09849196	11	403	2017	*aph(3')-III*	*erm(B)*	*tet(M)*
SAMN09849805	11	403	2017		*erm(A)*	*tet(M)*
SAMN09849626	11	403	2017		*erm(B)*	*tet(M)*
SAMN09849400	11	403	2017		*erm(B)*	*tet(M)*
SAMN09849376	11	403	2017		*erm(B)*	*tet(M)*
SAMN09849112	11	403	2017			
SAMN09849503	11	403	2017	*aph(3')-III*	*erm(B)*	*tet(M)*
SAMN09849128	11	403	2017	*aph(3')-III*	*erm(B)*	*tet(M)*
SAMN09849710	11	403	2017		*erm(A)*	*tet(M)*
SAMN09849763	11	403	2017			
SAMN09849760	11	403	2017			
SAMN09849762	11	403	2017		*erm(A)*	*tet(M)*
SAMN09849847	11	403	2017		*erm(A)*	*tet(M)*
SAMN09849877	11	403	2017		*erm(A)*	*tet(M)*
SAMN09849210	11	403	2017		*erm(B)*	*tet(M)*
SAMN09849301	11	403	2017		*erm(B)*	*tet(M)*
SAMN09849722	11	403	2018		*erm(A)*	*tet(M)*

SAMN09849767	11	403	2017		*erm(A)*	*tet(M)*
SAMN09849849	11	403	2017		*erm(A)*	*tet(M)*
SAMN09849901	11	403	2017			
SAMN09849565	11	403	2017			
SAMN07154009	75	49	2015			
SAMN07154008	75	49	2015			
SAMN07154006	75	49	2015			
SAMN07153882	75	49	2015			
SAMN07153580	75	150	2015			
SAMN07153581	75	861	2015		*mef(A) msr(D)*	
SAMN07153621	75	49	2015			
SAMN07153720	75	150	2015			
SAMN07153605	75	49	2015			
SAMN07153724	75	150	2015			
SAMN07153598	75	49	2015			
SAMN07153873	75	49	2015			
SAMN07153373	75	150	2015			
SAMN07153116	75	851	2015			
SAMN07153061	75	150	2015			
SAMN07153310	75	49	2015		*mef(A) msr(D)*	
SAMN07152970	75	49	2015			
SAMN07153361	75	49	2015		*mef(A) msr(D)*	
SAMN07153141	75	150	2015			
SAMN07153097	75	49	2015			
SAMN08690691	75	49	2016			
SAMN08691520	75	150	2016			
SAMN08691299	75	49	2016			
SAMN08691245	75	49	2016		*mef(A) msr(D)*	
SAMN08690561	75	150	2016		*erm(B)*	*tet(M)*
SAMN08690881	75	49	2016			
SAMN08691226	75	49	2016			
SAMN08690783	75	150	2016			
SAMN08690787	75	49	2016			
SAMN08691079	75	49	2016			
SAMN08691080	75	49	2016		*mef(A) msr(D)*	
SAMN08691083	75	861	2016		*mef(A) msr(D)*	
SAMN08691147	75	150	2016			
SAMN08690968	75	150	2016			
SAMN08691071	75	49	2016			
SAMN08691255	75	150	2016			
SAMN08690596	75	49	2016			
SAMN08690940	75	49	2016		*mef(A) msr(D)*	
SAMN08690794	75	49	2016			
SAMN08690967	75	150	2016			
SAMN08690578	75	150	2016			
SAMN08690538	75	49	2015			
SAMN08690937	75	150	2016			
SAMN08690907	75	49	2016			
SAMN08690767	75	861	2016		*mef(A) msr(D)*	
SAMN08691453	75	49	2016			
SAMN08691481	75	49	2016			
SAMN08692456	75	49	2017		*mef(A) msr(D)*	
SAMN08691729	75	150	2016			
SAMN08691730	75	150	2016			
SAMN08691731	75	150	2016			
SAMN08692244	75	49	2017		*mef(A) msr(D)*	
SAMN08692061	75	49	2016			
SAMN08691524	75	150	2016			
SAMN08691933	75	150	2016			
SAMN08692408	75	49	2017			
SAMN08692149	75	49	2016			
SAMN08691986	75	150	2016			
SAMN08691628	75	49	2016			
SAMN08692779	75	49	2017			
SAMN08692775	75	49	2017			
SAMN08692613	75	49	2017		*mef(A) msr(D)*	

SAMN08692546	75	49	2017		*mef(A) msr(D)*	
SAMN08692804	75	150	2017		*erm(B)*	*tet(M)*
SAMN08692645	75	49	2017			
SAMN08692655	75	49	2017			
SAMN08692762	75	49	2017			
SAMN08692911	75	49	2017			
SAMN08693160	75	150	2017		*erm(B)*	*tet(M)*
SAMN08692968	75	49	2017		*mef(A) msr(D)*	
SAMN08692891	75	150	2017			
SAMN08693024	75	150	2017			
SAMN08692834	75	1051	2017			
SAMN08692992	75	49	2017		*mef(A) msr(D)*	
SAMN09849527	75	150	2017		*erm(B)*	*tet(M)*
SAMN09849144	75	150	2017		*erm(B)*	*tet(M)*
SAMN09849606	75	150	2017			
SAMN09849610	75	49	2017			
SAMN09849776	75	150	2017			
SAMN09849234	75	150	2017		*erm(B)*	*tet(M)*
SAMN09849618	75	150	2017		*erm(B)*	*tet(M)*
SAMN09849622	75	150	2017		*erm(B)*	*tet(M)*
SAMN09849490	75	49	2017			
SAMN09848999	75	49	2017			
SAMN09849739	75	150	2017		*erm(B)*	*tet(M)*
SAMN09849260	75	49	2017			
SAMN09849297	75	49	2017			
SAMN09848995	75	49	2017			
SAMN07154225	77	133	2015			*tet(M)*
SAMN07154226	77	133	2015			*tet(M)*
SAMN07154187	77	63	2015		*erm(A)*	*tet(O)*
SAMN07154188	77	399	2015		*erm(T)*	*tet(M)*
SAMN07154190	77	63	2015		*erm(A)*	*tet(O)*
SAMN07154004	77	63	2015		*erm(A)*	*tet(O)*
SAMN07154081	77	399	2015		*erm(T)*	
SAMN07154082	77	63	2015			*tet(O)*
SAMN07154113	77	63	2015		*erm(A)*	*tet(O)*
SAMN07154218	77	63	2015		*erm(A)*	*tet(O)*
SAMN07154424	77	133	2015			*tet(M)*
SAMN07154088	77	63	2015		*erm(T)*	*tet(O)*
SAMN07154039	77	904	2015			*tet(M)*
SAMN07154123	77	399	2015			*tet(M)*
SAMN07154233	77	133	2015			*tet(M)*
SAMN07154254	77	63	2015		*erm(A)*	*tet(O)*
SAMN07154320	77	63	2015			*tet(O)*
SAMN07153667	77	63	2015		*erm(A)*	*tet(O)*
SAMN07153577	77	133	2015			*tet(M)*
SAMN07153486	77	63	2015			*tet(O)*
SAMN07153786	77	399	2015			*tet(M)*
SAMN07153749	77	63	2015			*tet(O)*
SAMN07153965	77	63	2015		*erm(A)*	*tet(O)*
SAMN07153655	77	63	2015		*erm(A)*	*tet(O)*
SAMN07153820	77	399	2015	*aph(3')-III*	*erm(B)*	*tet(M)*
SAMN07153927	77	63	2015		*erm(A)*	*tet(O)*
SAMN07153572	77	133	2015			*tet(M)*
SAMN07153571	77	133	2015			*tet(M)*
SAMN07153570	77	63	2015		*erm(A)*	*tet(O)*
SAMN07153726	77	399	2015		*erm(T)*	*tet(M)*
SAMN07153733	77	399	2015	*aph(3')-III*	*erm(B)*	*tet(M)*
SAMN07153898	77	63	2015		*erm(A)*	*tet(O)*
SAMN07153692	77	63	2015			*tet(O)*
SAMN07153652	77	399	2015		*erm(T)*	*tet(M)*
SAMN07153940	77	63	2015			*tet(O)*
SAMN07153812	77	63	2015		*erm(A)*	*tet(O)*
SAMN07153516	77	63	2015		*erm(A)*	*tet(O)*
SAMN07153640	77	63	2015		*erm(A)*	*tet(O)*
SAMN07153735	77	63	2015			
SAMN07153868	77	63	2015		*erm(A)*	*tet(O)*

SAMN07153685	77	63	2015			*tet(O)*
SAMN07153629	77	63	2015			*tet(O)*
SAMN07153628	77	63	2015			*tet(O)*
SAMN07153328	77	63	2015		*erm(A)*	*tet(O)*
SAMN07153442	77	63	2015			*tet(O)*
SAMN07153145	77	399	2015		*erm(T)*	*tet(M)*
SAMN07153148	77	399	2015		*erm(T)*	*tet(M)*
SAMN07153390	77	63	2015			*tet(O)*
SAMN07153457	77	63	2015			*tet(O)*
SAMN07153269	77	63	2015			*tet(O)*
SAMN07153359	77	399	2015		*erm(T)*	*tet(M)*
SAMN07153235	77	63	2015		*erm(A)*	*tet(O)*
SAMN07153452	77	63	2015			*tet(O)*
SAMN07153009	77	63	2015			
SAMN08690618	77	63	2016		*erm(A)*	*tet(O)*
SAMN08691506	77	63	2016		*erm(A)*	*tet(O)*
SAMN08690566	77	399	2016		*erm(T)*	
SAMN08691225	77	63	2016			*tet(O)*
SAMN08691218	77	63	2016			*tet(O)*
SAMN08690856	77	63	2016		*erm(A)*	*tet(O)*
SAMN08690920	77	63	2016		*erm(A)*	*tet(O)*
SAMN08691409	77	63	2016		*erm(A)*	*tet(O)*
SAMN08690664	77	133	2016			*tet(M)*
SAMN08691372	77	399	2016		*erm(T)*	*tet(M)*
SAMN08691385	77	63	2016		*erm(T)*	*tet(O)*
SAMN08691404	77	63	2016			*tet(O)*
SAMN08691178	77	63	2016			
SAMN08691185	77	63	2016			*tet(O)*
SAMN08690582	77	63	2016			*tet(O)*
SAMN08691336	77	63	2016		*erm(A)*	*tet(O)*
SAMN08691484	77	63	2016		*erm(A)*	*tet(O)*
SAMN08690906	77	63	2016		*erm(A)*	*tet(O)*
SAMN08690852	77	63	2016		*erm(A)*	*tet(O)*
SAMN08691216	77	63	2016			*tet(O)*
SAMN08690872	77	63	2016			
SAMN08691356	77	63	2016			*tet(O)*
SAMN08691490	77	133	2016			*tet(M)*
SAMN08691273	77	399	2016		*erm(T)*	*tet(M)*
SAMN08690710	77	63	2016		*erm(A)*	*tet(O)*
SAMN08691138	77	63	2016			*tet(O)*
SAMN08692510	77	63	2017			*tet(O)*
SAMN08691588	77	63	2016			*tet(O)*
SAMN08692230	77	399	2017			*tet(M)*
SAMN08692460	77	63	2017			*tet(O)*
SAMN08691885	77	63	2016			*tet(O)*
SAMN08691891	77	63	2016		*erm(A)*	*tet(O)*
SAMN08691893	77	63	2016			*tet(O)*
SAMN08692080	77	399	2016		*erm(T)*	*tet(M)*
SAMN08692076	77	399	2016			*tet(M)*
SAMN08691712	77	63	2016			*tet(O)*
SAMN08691705	77	63	2016		*erm(A)*	*tet(O)*
SAMN08691709	77	63	2016		*erm(A)*	*tet(O)*
SAMN08692158	77	63	2017			*tet(O)*
SAMN08692163	77	63	2017			*tet(O)*
SAMN08692237	77	63	2017		*erm(A)*	*tet(O)*
SAMN08692094	77	399	2016		*erm(T)*	*tet(M)*
SAMN08691701	77	63	2016		*erm(A)*	*tet(O)*
SAMN08691533	77	399	2016		*erm(T)*	*tet(M)*
SAMN08691929	77	63	2016		*erm(A)*	*tet(O)*
SAMN08691903	77	63	2016			*tet(O)*
SAMN08692409	77	399	2017			*tet(M)*
SAMN08692280	77	399	2017		*erm(T)*	*tet(M)*
SAMN08691721	77	133	2016			*tet(M)*
SAMN08691755	77	399	2016			*tet(M)*
SAMN08691757	77	399	2016			*tet(M)*
SAMN08691967	77	63	2016			*tet(O)*

SAMN08691664	77	63	2016		*erm(A)*	*tet(O)*
SAMN08691670	77	63	2016		*erm(A)*	*tet(O)*
SAMN08692352	77	63	2016		*erm(A)*	*tet(O)*
SAMN08692006	77	63	2016			*tet(O)*
SAMN08692143	77	63	2016			*tet(O)*
SAMN08692481	77	63	2017			*tet(O)*
SAMN08691994	77	63	2016		*erm(A)*	*tet(O)*
SAMN08691922	77	63	2016			
SAMN08692309	77	63	2017		*erm(A)*	*tet(O)*
SAMN08692449	77	63	2017			*tet(O)*
SAMN08692445	77	63	2017			*tet(O)*
SAMN08692035	77	63	2017			*tet(O)*
SAMN08692130	77	399	2016		*erm(T)*	*tet(M)*
SAMN08691736	77	482	2016			*tet(M)*
SAMN08691985	77	63	2016		*erm(A)*	*tet(O)*
SAMN08692068	77	399	2016			*tet(M)*
SAMN08691564	77	399	2016		*erm(T)*	*tet(M)*
SAMN08691560	77	63	2016			*tet(O)*
SAMN08692493	77	63	2017			*tet(O)*
SAMN08692566	77	63	2017			
SAMN08692683	77	399	2017		*erm(T)*	*tet(M)*
SAMN08692697	77	399	2017			*tet(M)*
SAMN08692802	77	63	2017		*erm(A)*	*tet(O)*
SAMN08692726	77	63	2016			*tet(O)*
SAMN08692571	77	63	2017		*erm(A)*	*tet(O)*
SAMN08692574	77	63	2017			
SAMN08693268	77	63	2017		*erm(A)*	*tet(O)*
SAMN08693112	77	63	2017			*tet(M)*
SAMN08693110	77	63	2017		*erm(A)*	*tet(O)*
SAMN08693044	77	399	2017			*tet(M)*
SAMN08693002	77	63	2017		*erm(A)*	*tet(O)*
SAMN08693005	77	63	2017		*erm(A)*	*tet(O)*
SAMN08692886	77	63	2017		*erm(A)*	*tet(O)*
SAMN08693056	77	399	2017			*tet(M)*
SAMN08693057	77	63	2017		*erm(A)*	*tet(O)*
SAMN08693129	77	63	2017		*erm(A)*	*tet(O)*
SAMN08693126	77	63	2017			*tet(O)*
SAMN08692995	77	63	2017		*erm(A)*	*tet(O)*
SAMN08693227	77	63	2016			*tet(O)*
SAMN08692867	77	63	2017			*tet(O)*
SAMN08693062	77	63	2017		*erm(A)*	*tet(O)*
SAMN08693103	77	63	2017			*tet(O)*
SAMN08693204	77	63	2017			*tet(O)*
SAMN09849186	77	399	2017		*erm(T)*	*tet(M)*
SAMN09849869	77	63	2017			*tet(O)*
SAMN09849871	77	63	2017		*erm(A)*	*tet(O)*
SAMN09849787	77	63	2017			*tet(O)*
SAMN09849444	77	399	2017			*tet(M)*
SAMN09849441	77	63	2017	*ant(6)-Ia*	*erm(A)*	*tet(O)*
SAMN09848975	77	63	2017			*tet(O)*
SAMN09849313	77	63	2017			*tet(O)*
SAMN09849163	77	63	2017	*ant(6)-Ia*	*erm(A)*	*tet(O)*
SAMN09849162	77	63	2017		*erm(A)*	*tet(O)*
SAMN09849609	77	399	2017		*erm(T)*	
SAMN09849207	77	63	2017		*erm(A)*	*tet(O)*
SAMN09849481	77	399	2017			*tet(M)*
SAMN09849677	77	399	2017		*erm(T)*	*tet(M)*
SAMN09849878	77	63	2017			*tet(O)*
SAMN09848992	77	399	2016		*erm(T)*	*tet(M)*
SAMN09849523	77	63	2017		*erm(A)*	*tet(O)*
SAMN09848926	77	399	2016		*erm(T)*	*tet(M)*
SAMN09849335	77	63	2017		*erm(A)*	*tet(O)*
SAMN09849644	77	63	2017		*erm(A)*	*tet(O)*
SAMN09849693	77	63	2017		*erm(A)*	*tet(O)*
SAMN09849349	77	63	2017		*erm(A)*	*tet(O)*
SAMN09849472	77	63	2017		*erm(A)*	*tet(O)*

SAMN09849666	77	63	2017		*erm(A)*	*tet(O)*
SAMN09849672	77	399	2017			*tet(M)*
SAMN09848924	77	63	2016			*tet(O)*
SAMN09849067	77	63	2017		*erm(A)*	*tet(O)*
SAMN09849402	77	63	2017		*erm(A)*	*tet(O)*
SAMN09849284	77	63	2017			*tet(O)*
SAMN09849291	77	63	2017		*erm(A)*	*tet(O)*
SAMN09849288	77	63	2017			*tet(O)*
SAMN09849359	77	63	2017		*erm(A)*	*tet(O)*
SAMN09849362	77	63	2017		*erm(A)*	*tet(O)*
SAMN09849262	77	63	2017			*tet(O)*
SAMN09849624	77	63	2017			*tet(O)*
SAMN09849390	77	63	2017		*erm(A)*	*tet(O)*
SAMN09849038	77	63	2017		*erm(A)*	*tet(O)*
SAMN09849041	77	63	2017			*tet(O)*
SAMN09849211	77	63	2017			
SAMN09849769	77	63	2017			*tet(O)*
SAMN07154285	92	82	2015	*ant(6)-Ia, aph(3')-III*	*erm(T)*	*tet(M)*
SAMN07154361	92	82	2015	*ant(6)-Ia, aph(3')-III*	*erm(T)*	*tet(M)*
SAMN07154390	92	82	2015	*ant(6)-Ia, aph(3')-III*	*erm(T)*	*tet(M)*
SAMN07154077	92	82	2015	*ant(6)-Ia, aph(3')-III*	*erm(T)*	*tet(M)*
SAMN07154079	92	82	2015	*ant(6)-Ia, aph(3')-III*	*erm(T)*	*tet(M)*
SAMN07154332	92	82	2015	*ant(6)-Ia, aph(3')-III*	*erm(T)*	*tet(M)*
SAMN07154330	92	82	2015	*ant(6)-Ia, aph(3')-III*	*erm(T)*	*tet(M)*
SAMN07154114	92	82	2015	*ant(6)-Ia, aph(3')-III*	*erm(T)*	*tet(M)*
SAMN07154214	92	82	2015	*ant(6)-Ia, aph(3')-III*	*erm(A)*	*tet(M)*
SAMN07154264	92	82	2015	*ant(6)-Ia, aph(3')-III*	*erm(T)*	*tet(M)*
SAMN07154265	92	82	2015	*ant(6)-Ia, aph(3')-III*	*erm(T)*	*tet(M)*
SAMN07154087	92	82	2015	*ant(6)-Ia, aph(3')-III*	*erm(T)*	*tet(M)*
SAMN07154091	92	82	2015	*ant(6)-Ia, aph(3')-III*	*erm(T)*	*tet(M)*
SAMN07154040	92	82	2015	*ant(6)-Ia, aph(3')-III*		*tet(M)*
SAMN07154247	92	82	2015	*ant(6)-Ia, aph(3')-III*	*erm(T)*	*tet(M)*
SAMN07154252	92	82	2015	*ant(6)-Ia, aph(3')-III*	*erm(T)*	*tet(M)*
SAMN07154096	92	82	2015	*ant(6)-Ia, aph(3')-III*	*erm(T)*	*tet(M)*
SAMN07154262	92	82	2015	*ant(6)-Ia, aph(3')-III*	*erm(T)*	*tet(M)*
SAMN07154275	92	82	2015	*ant(6)-Ia, aph(3')-III*		*tet(M)*
SAMN07154024	92	82	2015	*ant(6)-Ia, aph(3')-III*	*erm(T)*	*tet(M)*
SAMN07154027	92	82	2015	*ant(6)-Ia, aph(3')-III*	*erm(T)*	*tet(M)*
SAMN07154235	92	82	2015	*ant(6)-Ia, aph(3')-III*	*erm(T)*	*tet(M)*
SAMN07153979	92	82	2015	*ant(6)-Ia, aph(3')-III*	*erm(T)*	*tet(M)*
SAMN07154104	92	82	2015	*ant(6)-Ia, aph(3')-III*	*erm(T)*	*tet(M)*
SAMN07153881	92	82	2015	*ant(6)-Ia, aph(3')-III*	*erm(T)*	*tet(M)*
SAMN07153785	92	82	2015	*ant(6)-Ia, aph(3')-III*	*erm(T)*	*tet(M)*
SAMN07153721	92	82	2015	*ant(6)-Ia, aph(3')-III*	*erm(T)*	*tet(M)*
SAMN07153718	92	82	2015	*ant(6)-Ia, aph(3')-III*		*tet(M)*
SAMN07153892	92	82	2015	*ant(6)-Ia, aph(3')-III*	*erm(T)*	*tet(M)*
SAMN07153886	92	82	2015	*ant(6)-Ia, aph(3')-III*	*erm(T)*	*tet(M)*
SAMN07153750	92	82	2015			
SAMN07153777	92	82	2015	*ant(6)-Ia, aph(3')-III*	*erm(T)*	*tet(M)*
SAMN07153896	92	82	2015	*ant(6)-Ia, aph(3')-III*	*erm(T)*	*tet(M)*
SAMN07153805	92	82	2015	*ant(6)-Ia, aph(3')-III*	*erm(T)*	*tet(M)*
SAMN07153765	92	82	2015	*ant(6)-Ia, aph(3')-III*	*erm(T)*	*tet(M)*
SAMN07153764	92	82	2015	*ant(6)-Ia, aph(3')-III*	*erm(T)*	*tet(M)*
SAMN07153737	92	82	2015	*ant(6)-Ia, aph(3')-III*	*erm(T)*	*tet(M)*
SAMN07153549	92	82	2015	*ant(6)-Ia, aph(3')-III*	*erm(T)*	*tet(M)*
SAMN07153942	92	82	2015	*ant(6)-Ia, aph(3')-III*	*erm(T)*	*tet(M)*
SAMN07153801	92	82	2015	*ant(6)-Ia, aph(3')-III*	*erm(A), erm(T)*	*tet(M)*
SAMN07153955	92	82	2015	*ant(6)-Ia, aph(3')-III*	*erm(T)*	*tet(M)*
SAMN07153953	92	82	2015	*ant(6)-Ia, aph(3')-III*	*erm(T)*	*tet(M)*
SAMN07153954	92	82	2015	*ant(6)-Ia, aph(3')-III*	*erm(T)*	*tet(M)*
SAMN07153192	92	82	2015	*ant(6)-Ia, aph(3')-III*	*erm(T)*	*tet(M)*
SAMN07153226	92	82	2015	*ant(6)-Ia, aph(3')-III*	*erm(T)*	*tet(M)*
SAMN07153228	92	82	2015	*ant(6)-Ia, aph(3')-III*	*erm(T)*	*tet(M)*
SAMN07153407	92	82	2015	*ant(6)-Ia, aph(3')-III*	*erm(T)*	*tet(M)*
SAMN07153245	92	855	2015	*ant(6)-Ia, aph(3')-III*	*erm(T)*	*tet(M)*
SAMN07153387	92	82	2015	*ant(6)-Ia, aph(3')-III*	*erm(T)*	*tet(M)*

SAMN07153389	92	82	2015	*ant(6)-Ia, aph(3')-III*	*erm(T)*	*tet(M)*
SAMN07153205	92	82	2015	*ant(6)-Ia, aph(3')-III*	*erm(T)*	*tet(M)*
SAMN07153000	92	82	2015	*ant(6)-Ia, aph(3')-III*	*erm(T)*	*tet(M)*
SAMN07153308	92	82	2015	*ant(6)-Ia, aph(3')-III*	*erm(T)*	*tet(M)*
SAMN07153414	92	82	2015	*ant(6)-Ia, aph(3')-III*	*erm(T)*	*tet(M)*
SAMN07153243	92	82	2015	*ant(6)-Ia, aph(3')-III*	*erm(T)*	*tet(M)*
SAMN07153244	92	82	2015	*ant(6)-Ia, aph(3')-III*	*erm(T)*	*tet(M)*
SAMN07153136	92	82	2015	*ant(6)-Ia, aph(3')-III*	*erm(T)*	*tet(M)*
SAMN07153031	92	82	2015	*ant(6)-Ia, aph(3')-III*	*erm(T)*	*tet(M)*
SAMN08691283	92	82	2016	*ant(6)-Ia, aph(3')-III*	*erm(T)*	*tet(M)*
SAMN08690655	92	82	2016	*ant(6)-Ia, aph(3')-III*	*erm(T)*	*tet(M)*
SAMN08690721	92	82	2016	*ant(6)-Ia, aph(3')-III*	*erm(T)*	*tet(M)*
SAMN08691389	92	82	2016	*ant(6)-Ia, aph(3')-III*	*erm(T)*	*tet(M)*
SAMN08691166	92	82	2016	*ant(6)-Ia, aph(3')-III*	*erm(T)*	*tet(M)*
SAMN08691120	92	82	2016	*ant(6)-Ia, aph(3')-III*	*erm(T)*	*tet(M)*
SAMN08691470	92	82	2016	*ant(6)-Ia, aph(3')-III*	*erm(T)*	*tet(M)*
SAMN08691100	92	82	2016	*ant(6)-Ia, aph(3')-III*	*erm(T)*	*tet(M)*
SAMN08691135	92	82	2016	*ant(6)-Ia, aph(3')-III*	*erm(T)*	*tet(M)*
SAMN08691129	92	82	2016	*ant(6)-Ia, aph(3')-III*	*erm(T)*	*tet(M)*
SAMN08690637	92	82	2016	*ant(6)-Ia, aph(3')-III*	*erm(T)*	*tet(M)*
SAMN08690631	92	82	2016	*ant(6)-Ia, aph(3')-III*	*erm(T)*	*tet(M)*
SAMN08691153	92	82	2016	*ant(6)-Ia, aph(3')-III*	*erm(T)*	*tet(M)*
SAMN08691293	92	82	2016	*ant(6)-Ia, aph(3')-III*	*erm(T)*	*tet(M)*
SAMN08690625	92	82	2016	*ant(6)-Ia, aph(3')-III*	*erm(T)*	*tet(M)*
SAMN08690572	92	82	2016	*ant(6)-Ia, aph(3')-III*	*erm(T)*	*tet(M)*
SAMN08692318	92	82	2017	*ant(6)-Ia, aph(3')-III*	*erm(T)*	*tet(M)*
SAMN08691589	92	82	2016	*ant(6)-Ia, aph(3')-III*	*erm(T)*	*tet(M)*
SAMN08692336	92	82	2016	*ant(6)-Ia, aph(3')-III*	*erm(T)*	*tet(M)*
SAMN08692227	92	82	2017	*ant(6)-Ia, aph(3')-III*	*erm(T)*	*tet(M)*
SAMN08691693	92	82	2016	*ant(6)-Ia, aph(3')-III*	*erm(T)*	*tet(M)*
SAMN08692188	92	82	2016	*ant(6)-Ia, aph(3')-III*	*erm(T)*	*tet(M)*
SAMN08691819	92	82	2016	*ant(6)-Ia, aph(3')-III*	*erm(T)*	*tet(M)*
SAMN08692338	92	82	2016	*ant(6)-Ia, aph(3')-III*	*erm(T)*	*tet(M)*
SAMN08691844	92	82	2016	*ant(6)-Ia, aph(3')-III*	*erm(T)*	*tet(M)*
SAMN08691983	92	82	2016	*ant(6)-Ia, aph(3')-III*	*erm(T)*	*tet(M)*
SAMN08691824	92	82	2016	*ant(6)-Ia, aph(3')-III*	*erm(T)*	*tet(M)*
SAMN08691831	92	82	2016	*ant(6)-Ia, aph(3')-III*	*erm(T)*	*tet(M)*
SAMN08692317	92	82	2017	*ant(6)-Ia, aph(3')-III*	*erm(T)*	*tet(M)*
SAMN08691697	92	82	2016	*ant(6)-Ia, aph(3')-III*	*erm(T)*	*tet(M)*
SAMN08692361	92	82	2016	*ant(6)-Ia, aph(3')-III*	*erm(T)*	*tet(M)*
SAMN08691806	92	82	2016	*ant(6)-Ia, aph(3')-III*	*erm(T)*	*tet(M)*
SAMN08691536	92	82	2016	*ant(6)-Ia, aph(3')-III*	*erm(T)*	*tet(M)*
SAMN08692217	92	82	2017	*ant(6)-Ia, aph(3')-III*	*erm(T)*	*tet(M)*
SAMN08692215	92	82	2017	*ant(6)-Ia, aph(3')-III*	*erm(T)*	*tet(M)*
SAMN08692219	92	82	2017	*ant(6)-Ia, aph(3')-III*	*erm(T)*	*tet(M)*
SAMN08691718	92	82	2016	*ant(6)-Ia, aph(3')-III*	*erm(T)*	*tet(M)*
SAMN08691965	92	82	2016	*ant(6)-Ia, aph(3')-III*	*erm(T)*	*tet(M)*
SAMN08692368	92	82	2016	*ant(6)-Ia, aph(3')-III*	*erm(T)*	*tet(M)*
SAMN08691790	92	82	2016	*ant(6)-Ia, aph(3')-III*	*erm(T)*	*tet(M)*
SAMN08692345	92	82	2016	*ant(6)-Ia, aph(3')-III*	*erm(T)*	*tet(M)*
SAMN08691963	92	82	2016	*ant(6)-Ia, aph(3')-III*	*erm(T)*	*tet(M)*
SAMN08692011	92	82	2016	*ant(6)-Ia, aph(3')-III*	*erm(T)*	*tet(M)*
SAMN08692007	92	82	2016	*ant(6)-Ia, aph(3')-III*	*erm(T)*	*tet(M)*
SAMN08692206	92	82	2016	*ant(6)-Ia, aph(3')-III*	*erm(T)*	*tet(M)*
SAMN08692378	92	82	2016	*ant(6)-Ia, aph(3')-III*	*erm(T)*	*tet(M)*
SAMN08691873	92	82	2016	*ant(6)-Ia, aph(3')-III*	*erm(T)*	*tet(M)*
SAMN08692436	92	82	2017	*ant(6)-Ia, aph(3')-III*	*erm(T)*	*tet(M)*
SAMN08692008	92	82	2016	*ant(6)-Ia, aph(3')-III*	*erm(T)*	*tet(M)*
SAMN08692313	92	82	2017	*ant(6)-Ia, aph(3')-III*	*erm(T)*	*tet(M)*
SAMN08692307	92	82	2017	*ant(6)-Ia, aph(3')-III*	*erm(T)*	*tet(M)*
SAMN08692194	92	82	2016	*ant(6)-Ia, aph(3')-III*	*erm(T)*	*tet(M)*
SAMN08692392	92	82	2016	*ant(6)-Ia, aph(3')-III*	*erm(T)*	*tet(M)*
SAMN08692325	92	82	2017	*ant(6)-Ia, aph(3')-III*	*erm(T)*	*tet(M)*
SAMN08691876	92	82	2016	*ant(6)-Ia, aph(3')-III*	*erm(T)*	*tet(M)*
SAMN08691989	92	82	2016	*ant(6)-Ia, aph(3')-III*	*erm(T)*	*tet(M)*
SAMN08691624	92	82	2016	*ant(6)-Ia, aph(3')-III*	*erm(T)*	*tet(M)*

SAMN08691562	92	82	2016	*ant(6)-Ia, aph(3')-III*	*erm(T)*	*tet(M)*
SAMN08692394	92	82	2016	*ant(6)-Ia, aph(3')-III*	*erm(T)*	*tet(M)*
SAMN08692561	92	82	2017	*ant(6)-Ia, aph(3')-III*	*erm(T)*	*tet(M)*
SAMN08692557	92	82	2017	*ant(6)-Ia, aph(3')-III*	*erm(T)*	*tet(M)*
SAMN08692758	92	82	2017	*ant(6)-Ia, aph(3')-III*	*erm(T)*	*tet(M)*
SAMN08692550	92	82	2017	*ant(6)-Ia, aph(3')-III*	*erm(T)*	*tet(M)*
SAMN08692792	92	82	2017	*ant(6)-Ia, aph(3')-III*	*erm(T)*	*tet(M)*
SAMN08692617	92	82	2017	*ant(6)-Ia, aph(3')-III*	*erm(T)*	*tet(M)*
SAMN08692808	92	82	2017	*ant(6)-Ia, aph(3')-III*	*erm(T)*	*tet(M)*
SAMN08692806	92	82	2017	*ant(6)-Ia, aph(3')-III*	*erm(T)*	*tet(M)*
SAMN08692736	92	82	2017	*ant(6)-Ia, aph(3')-III*	*erm(T)*	*tet(M)*
SAMN08692712	92	82	2016	*ant(6)-Ia, aph(3')-III*	*erm(T)*	*tet(M)*
SAMN08692755	92	82	2017	*ant(6)-Ia, aph(3')-III*	*erm(T)*	*tet(M)*
SAMN08692753	92	82	2017	*ant(6)-Ia, aph(3')-III*	*erm(T)*	*tet(M)*
SAMN08692580	92	82	2017	*ant(6)-Ia, aph(3')-III*	*erm(T)*	*tet(M)*
SAMN08692761	92	82	2017	*ant(6)-Ia, aph(3')-III*	*erm(T)*	*tet(M)*
SAMN08692889	92	82	2017	*ant(6)-Ia, aph(3')-III*	*erm(T)*	*tet(M)*
SAMN08693162	92	82	2017	*ant(6)-Ia, aph(3')-III*	*erm(T)*	*tet(M)*
SAMN08693033	92	82	2017	*ant(6)-Ia, aph(3')-III*	*erm(T)*	*tet(M)*
SAMN08692932	92	82	2017	*ant(6)-Ia, aph(3')-III*	*erm(T)*	*tet(M)*
SAMN08693076	92	82	2016	*ant(6)-Ia, aph(3')-III*	*erm(T)*	*tet(M)*
SAMN08693008	92	82	2017	*ant(6)-Ia, aph(3')-III*	*erm(T)*	*tet(M)*
SAMN08693009	92	82	2017	*ant(6)-Ia, aph(3')-III*	*erm(T)*	*tet(M)*
SAMN08693001	92	82	2017	*ant(6)-Ia, aph(3')-III*	*erm(T)*	*tet(M)*
SAMN08693121	92	82	2017	*ant(6)-Ia, aph(3')-III*	*erm(T)*	*tet(M)*
SAMN08692929	92	82	2017	*ant(6)-Ia, aph(3')-III*	*erm(T)*	*tet(M)*
SAMN08692926	92	82	2017	*ant(6)-Ia, aph(3')-III*	*erm(T)*	*tet(M)*
SAMN08692927	92	82	2017	*ant(6)-Ia, aph(3')-III*	*erm(T)*	*tet(M)*
SAMN08692997	92	82	2017		*erm(A)*	
SAMN08693245	92	82	2017	*ant(6)-Ia, aph(3')-III*	*erm(T)*	*tet(M)*
SAMN08693259	92	82	2017	*ant(6)-Ia, aph(3')-III*	*erm(T)*	*tet(M)*
SAMN08693106	92	82	2017	*ant(6)-Ia, aph(3')-III*	*erm(T)*	*tet(M)*
SAMN09849118	92	82	2017	*ant(6)-Ia, aph(3')-III*	*erm(T)*	*tet(M)*
SAMN09849117	92	82	2017	*ant(6)-Ia, aph(3')-III*	*erm(T)*	*tet(M)*
SAMN09849116	92	82	2017	*ant(6)-Ia, aph(3')-III*	*erm(T)*	*tet(M)*
SAMN09849120	92	82	2017	*ant(6)-Ia, aph(3')-III*	*erm(T)*	*tet(M)*
SAMN09849856	92	82	2017	*ant(6)-Ia, aph(3')-III*	*erm(T)*	*tet(M)*
SAMN09849752	92	82	2017	*ant(6)-Ia, aph(3')-III*	*erm(T)*	*tet(M)*
SAMN09849750	92	82	2017	*ant(6)-Ia, aph(3')-III*	*erm(T)*	*tet(M)*
SAMN09849416	92	82	2017	*ant(6)-Ia, aph(3')-III*	*erm(T)*	*tet(M)*
SAMN09849424	92	82	2017	*ant(6)-Ia, aph(3')-III*	*erm(T)*	*tet(M)*
SAMN09849596	92	82	2017	*ant(6)-Ia, aph(3')-III*	*erm(T)*	*tet(M)*
SAMN09849221	92	82	2017	*ant(6)-Ia, aph(3')-III*	*erm(T)*	*tet(M)*
SAMN09849219	92	82	2017	*ant(6)-Ia, aph(3')-III*	*erm(T)*	*tet(M)*
SAMN09849217	92	82	2017	*ant(6)-Ia, aph(3')-III*	*erm(T)*	*tet(M)*
SAMN09849550	92	82	2017	*ant(6)-Ia, aph(3')-III*	*erm(T)*	*tet(M)*
SAMN09849314	92	82	2017	*ant(6)-Ia, aph(3')-III*	*erm(T)*	*tet(M)*
SAMN09849552	92	82	2017	*ant(6)-Ia, aph(3')-III*	*erm(T)*	*tet(M)*
SAMN09849308	92	82	2017	*ant(6)-Ia, aph(3')-III*	*erm(T)*	*tet(M)*
SAMN09849312	92	82	2017	*ant(6)-Ia, aph(3')-III*	*erm(T)*	*tet(M)*
SAMN09849548	92	82	2017	*ant(6)-Ia, aph(3')-III*	*erm(T)*	*tet(M)*
SAMN09849581	92	82	2017	*ant(6)-Ia, aph(3')-III*	*erm(T)*	*tet(M)*
SAMN09849855	92	82	2017	*ant(6)-Ia, aph(3')-III*	*erm(T)*	*tet(M)*
SAMN09849861	92	82	2017	*ant(6)-Ia, aph(3')-III*	*erm(T)*	*tet(M)*
SAMN09849862	92	82	2017	*ant(6)-Ia, aph(3')-III*	*erm(T)*	*tet(M)*
SAMN09849586	92	82	2017	*ant(6)-Ia, aph(3')-III*	*erm(T)*	*tet(M)*
SAMN09849227	92	82	2017	*ant(6)-Ia, aph(3')-III*	*erm(T)*	*tet(M)*
SAMN09849408	92	82	2017	*ant(6)-Ia, aph(3')-III*	*erm(T)*	*tet(M)*
SAMN09849830	92	82	2017	*ant(6)-Ia, aph(3')-III*	*erm(T)*	*tet(M)*
SAMN09848933	92	82	2016	*ant(6)-Ia, aph(3')-III*	*erm(T)*	*tet(M)*
SAMN09848932	92	82	2016	*ant(6)-Ia, aph(3')-III*	*erm(B), erm(T)*	
SAMN09849557	92	82	2017	*ant(6)-Ia, aph(3')-III*	*erm(T)*	*tet(M)*
SAMN09849411	92	82	2017	*ant(6)-Ia, aph(3')-III*	*erm(T)*	*tet(M)*
SAMN09849228	92	82	2017	*ant(6)-Ia, aph(3')-III*	*erm(T)*	*tet(M)*
SAMN09849226	92	82	2017	*ant(6)-Ia, aph(3')-III*	*erm(A), erm(T)*	*tet(M)*
SAMN09849225	92	82	2017	*ant(6)-Ia, aph(3')-III*	*erm(T)*	*tet(M)*

SAMN09849342	92	82	2017	*ant(6)-Ia, aph(3')-III*	*erm(T)*	*tet(M)*
SAMN09849339	92	82	2017	*ant(6)-Ia, aph(3')-III*	*erm(T)*	*tet(M)*
SAMN09849336	92	82	2017	*ant(6)-Ia, aph(3')-III*	*erm(T)*	*tet(M)*
SAMN09849842	92	82	2017	*ant(6)-Ia, aph(3')-III*	*erm(T)*	*tet(M)*
SAMN09849050	92	82	2017	*ant(6)-Ia, aph(3')-III*	*erm(T)*	*tet(M)*
SAMN09848968	92	82	2016	*ant(6)-Ia, aph(3')-III*	*erm(T)*	*tet(M)*
SAMN09849352	92	82	2017	*ant(6)-Ia, aph(3')-III*	*erm(T)*	*tet(M)*
SAMN09849345	92	82	2017		*erm(A)*	
SAMN09849353	92	82	2017	*ant(6)-Ia, aph(3')-III*	*erm(T)*	*tet(M)*
SAMN09849566	92	82	2017	*ant(6)-Ia, aph(3')-III*	*erm(T)*	*tet(M)*
SAMN09849670	92	82	2017	*ant(6)-Ia, aph(3')-III*	*erm(T)*	*tet(M)*
SAMN09849326	92	82	2017	*ant(6)-Ia, aph(3')-III*	*erm(T)*	*tet(M)*
SAMN09849330	92	82	2017	*ant(6)-Ia, aph(3')-III*	*erm(T)*	*tet(M)*
SAMN09849334	92	82	2017	*ant(6)-Ia, aph(3')-III*	*erm(T)*	*tet(M)*
SAMN09849095	92	82	2017	*ant(6)-Ia, aph(3')-III*	*erm(T)*	*tet(M)*
SAMN09849100	92	82	2017	*ant(6)-Ia, aph(3')-III*	*erm(T)*	*tet(M)*
SAMN09849101	92	82	2017	*ant(6)-Ia, aph(3')-III*	*erm(T)*	*tet(M)*
SAMN09849103	92	82	2017	*ant(6)-Ia, aph(3')-III*	*erm(T)*	*tet(M)*
SAMN09849104	92	82	2017	*ant(6)-Ia, aph(3')-III*	*erm(T)*	*tet(M)*
SAMN09849417	92	82	2017	*ant(6)-Ia, aph(3')-III*	*erm(T)*	*tet(M)*
SAMN09849418	92	82	2017	*ant(6)-Ia, aph(3')-III*	*erm(T)*	*tet(M)*
SAMN09849069	92	82	2017	*ant(6)-Ia, aph(3')-III*	*erm(T)*	*tet(M)*
SAMN09849890	92	82	2017	*ant(6)-Ia, aph(3')-III*	*erm(T)*	*tet(M)*
SAMN09849248	92	82	2017	*ant(6)-Ia, aph(3')-III*	*erm(T)*	*tet(M)*
SAMN09849247	92	82	2017	*ant(6)-Ia, aph(3')-III*	*erm(T)*	*tet(M)*
SAMN09849809	92	82	2017	*ant(6)-Ia, aph(3')-III*	*erm(T)*	*tet(M)*
SAMN09849059	92	82	2017		*erm(A)*	
SAMN09849058	92	82	2017	*ant(6)-Ia, aph(3')-III*	*erm(T)*	*tet(M)*
SAMN09848960	92	82	2016	*ant(6)-Ia, aph(3')-III*	*erm(T)*	*tet(M)*
SAMN09849564	92	82	2017	*ant(6)-Ia, aph(3')-III*	*erm(T)*	*tet(M)*
SAMN09849563	92	82	2017	*ant(6)-Ia, aph(3')-III*	*erm(T)*	*tet(M)*
SAMN09849851	92	82	2017	*ant(6)-Ia, aph(3')-III*	*erm(T)*	*tet(M)*
SAMN09849106	92	82	2017	*ant(6)-Ia, aph(3')-III*	*erm(T)*	*tet(M)*
SAMN09849108	92	82	2017	*ant(6)-Ia, aph(3')-III*	*erm(T)*	*tet(M)*
SAMN09849083	92	82	2017	*ant(6)-Ia, aph(3')-III*	*erm(T)*	*tet(M)*
SAMN09849129	92	82	2017	*ant(6)-Ia, aph(3')-III*	*erm(T)*	*tet(M)*
SAMN09849598	92	82	2017	*ant(6)-Ia, aph(3')-III*	*erm(T)*	*tet(M)*
SAMN09849197	92	82	2017	*ant(6)-Ia, aph(3')-III*	*erm(T)*	*tet(M)*
SAMN09849255	92	82	2017	*ant(6)-Ia, aph(3')-III*	*erm(T)*	*tet(M)*
SAMN09849817	92	82	2017	*ant(6)-Ia, aph(3')-III*	*erm(T)*	*tet(M)*
SAMN09849820	92	82	2017	*ant(6)-Ia, aph(3')-III*	*erm(T)*	*tet(M)*
SAMN09849638	92	82	2017	*ant(6)-Ia, aph(3')-III*	*erm(T)*	*tet(M)*
SAMN09849814	92	82	2017	*ant(6)-Ia, aph(3')-III*	*erm(T)*	*tet(M)*
SAMN09849430	92	82	2017	*ant(6)-Ia, aph(3')-III*	*erm(T)*	*tet(M)*
SAMN09849393	92	82	2017	*ant(6)-Ia, aph(3')-III*	*erm(T)*	*tet(M)*
SAMN09849426	92	82	2017	*ant(6)-Ia, aph(3')-III*	*erm(T)*	*tet(M)*
SAMN09849428	92	82	2017	*ant(6)-Ia, aph(3')-III*	*erm(T)*	*tet(M)*
SAMN09849427	92	82	2017	*ant(6)-Ia, aph(3')-III*	*erm(T)*	*tet(M)*
SAMN09849214	92	82	2017	*ant(6)-Ia, aph(3')-III*	*erm(T)*	*tet(M)*
SAMN09848967	92	82	2016	*ant(6)-Ia, aph(3')-III*	*erm(T)*	*tet(M)*
SAMN09849316	92	82	2017	*ant(6)-Ia, aph(3')-III*	*erm(T)*	*tet(M)*
SAMN09849319	92	82	2017	*ant(6)-Ia, aph(3')-III*	*erm(T)*	*tet(M)*
SAMN09849804	92	82	2017	*ant(6)-Ia, aph(3')-III*	*erm(T)*	*tet(M)*
SAMN09849770	92	82	2017	*ant(6)-Ia, aph(3')-III*	*erm(T)*	*tet(M)*
SAMN09849772	92	82	2017	*ant(6)-Ia, aph(3')-III*	*erm(T)*	*tet(M)*
SAMN09849573	92	82	2017	*ant(6)-Ia, aph(3')-III*	*erm(T)*	*tet(M)*
SAMN09849852	92	82	2017	*ant(6)-Ia, aph(3')-III*	*erm(T)*	*tet(M)*
SAMN09849574	92	82	2017	*ant(6)-Ia, aph(3')-III*	*erm(T)*	*tet(M)*

[a] Biosample number under BioProject PRJNA395240.

[b] GAS *emm* type determined as described in Methods.

[c] Sequence type (ST) determined as described in Methods.

[d] Year of isolation. Strains after 2015 are currently unpublished (B. Beall personal communication).

[e] Antibiotic resistance genes identified through bioinformatics pipeline defined in Methods for aminoglycosides (AG), macrolide-lincosamide-streptogramin (MLS), tetracycline (TET).

TABLE S3. PHE GAS strains used in this study.

Run Accession[a]	Emm[b]	ST[c]	Year[d]	AG[e]	MLS	TET
SAMEA3930755	75	150	2014			
SAMEA3930594	75	150	2014			
SAMEA3931077	75	150	2014			
SAMEA3930576	75	150	2014			
SAMEA3930750	75	150	2014			
SAMEA3930876	75	150	2014			
SAMEA3930769	75	150	2014			
SAMEA3931126	75	150	2014			
SAMEA3930802	75	150	2014			
SAMEA3931085	75	150	2014			
SAMEA3930746	75	150	2014			
SAMEA3930874	75	150	2014			
SAMEA3930785	75	150	2014			
SAMEA3930936	75	150	2014			
SAMEA3931116	75	150	2014			
SAMEA3930981	75	150	2014			
SAMEA3930863	75	150	2014			
SAMEA3930749	77	399	2014			*tet(M)*
SAMEA3930908	77	63	2014		*erm(A)*	*tet(O)*
SAMEA3931070	77	63	2014		*erm(A)*	*tet(O)*

[a] Biosample number under BioProject PRJEB13551.

[b] GAS *emm* type determined as described in Methods.

[c] Sequence type (ST) determined as described in Methods.

[d] Year of isolation.

[e] Antibiotic resistance genes identified through bioinformatics pipeline defined in Methods for aminoglycosides (AG), macrolide-lincosamide-streptogramin (MLS), tetracycline (TET).

TABLE S4. GAS *emm* types defined by high (>50%) and low intrinsic resistance.

emm	Invasive Index[a]	Total (%) (n=929)	Any Resistance (%)[b]	O/E[c] (Any)	TE Resistance (%)	O/E (TE)	M/MLS Resistance (%)	O/E (M/MLS)
**Low**								
1	1.41	199 (21.4)	1 (0.5)	0.09	1 (0.5	0.09	1 (0.5)	0.07
2	0.41	11 (1.2)	0 (0.0)	-	-	-	-	-
3	1.85	76 (8.2)	2 (2.6)	0.24	1 (1.3)	0.24	2 (2.6)	0.38
4	0.37	86 (9.3)	3 (3.5)	0.32	-	-	3 (3.5)	0.50
6	0.60	71 (7.6)	2 (2.8)	0.26	1 (1.4)	0.25	2 (2.8)	0.40
12	0.56	145 (15.6)	5 (3.5)	0.32	2 (1.4)	0.25	1 (0.7)	0.10
22	1.27	25 (2.7)	1 (4.0)	0.37	1 (4.0)	0.71	-	-
28	1.50	51 (5.5)	5 (9.8)	0.91	-	-	5 (9.8)	1.40
87	1.39	30 (3.2)	2 (6.7)	0.62	2 (6.7)	1.19	1 (3.3)	0.48
89	0.78	136 (14.6)	2 (1.5)	0.14	1 (0.7)	0.13	2 (1.5)	0.21
**Overall**	**0.96**	**830 (89.3)**	**23 (2.8)**	**0.26**	**9 (1.1)**	**0.19**	**17 (2.1)**	**0.29**
**High**								
11	4.92	11 (1.2)	9 (81.8)	7.60	9 (81.8)	14.62	7 (63.6)	9.10
75	1.84	17 (1.8)	11 (64.7)	6.01	2 (11.8)	2.10	9 (52.9)	7.57
77	1.64	22 (2.4)	22 (100)	9.29	22 (100)	17.87	7 (31.8)	4.55
92	NA[d]	11 (1.2)	11 (100)	9.29	11 (100)	17.87	11 (100)	14.29
**Overall**	**2.70***	**61 (6.6)**	**53 (86.8)**	**8.07****	**44 (72.1)**	**9.26****	**30 (49.2)**	**7.97****

[a] Invasive index (index) defined as the frequency of occurrence in invasive divided by the frequency of occurrence in pharyngeal diseases for each *emm* type and collectively by group (Overall).

[b] Percent resistance within the given *emm* type.

[c] Observed/Expected resistance (O/E) ratios defined in Methods.

[d] Invasive index not calculable for *emm92* as none identified in pharyngeal disease.

* *P*=0.03 and ** *P*=0.003 (Mann-Whitney U test) compared to Low.

TABLE S5. Summary of completed genomes for *emm11*, *emm75*, *emm77*, and *em92* GAS strains.

Strain (Accession no.)	*emm* type	Chromosome (bp)	Plasmid (bp)	Prophage[a]	Prophage-associated genes[b]	Other MGE[c]
TSPY155<br>(CP032699)	11	1882327	-	3	*speH1, speC2,*<br>*sdn/mf*	Tn*6002*<br>*tet(M), erm(B)*
TSPY165<br>(CP033336)	77	1812036	-	1	*speM5, sla*	Tn*916*<br>*tet(M)*
TSPY208<br>(CP033335)	75	1801696	-	4	*speK, speL1,*<br>*speC6, sdn/mf* (2)	Tn*1207.1*<br>*mef(A), msr(D)*
TSPY453<br>(CP033336)	77	1976011	-	3	*speK, sdn/mf* (3)	ICESpyM77.2<br>*tet(O), erm(A)*
TSPY556<br>(CP032700;<br>CP032701)	92	1828132	4968	1	*speK, sdn/mf*	ICESpyM92<br>*tet(M), aph(3')-III,*<br>*ant(6)-Ia*

[a] Prophage identified using Phaster (11).

[b] GAS virulence factors residing within prophage.

[c] Mobile genetic elements (MGE) identified using ISFinder (12), IslandViewer (13), and ICEBerg (14).

TABLE S6. Minimal inhibitory concentrations (MICs) to selected aminoglycosides for *emm11* and *emm92* GAS strains from Houston.

Strain	*emm* Type	AG Resistance Gene(s)	Kanamycin[1]	Streptomycin	Gentamicin
TSPY556	92	*ant(6)-Ia, aph(3')-III*	>2000	>2000	1.5
TSPY783	92	*ant(6)-Ia, aph(3')-III*	>2000	>2000	2.0
TSPY854	92	*ant(6)-Ia, aph(3')-III*	2000	>2000	2.0
TSPY1042	92	*ant(6)-Ia, aph(3')-III*	>2000	>2000	1.5
TSPY1076	92	*ant(6)-Ia, aph(3')-III*	1000	1000	1.5
TSPY1089	92	*ant(6)-Ia, aph(3')-III*	>2000	>2000	2.0
TSPY1195	92	*ant(6)-Ia, aph(3')-III*	2000	2000	3.0
TSPY1218	92	*ant(6)-Ia, aph(3')-III*	>2000	>2000	1.5
TSPY1227	92	*ant(6)-Ia, aph(3')-III*	2000	2000	1.5
TSPY1285	92	*ant(6)-Ia, aph(3')-III*	>2000	>2000	1.5
TSPY1287	92	*ant(6)-Ia, aph(3')-III*	>2000	>2000	0.75
TSPY155	11		62.5	15.6	2.0
TSPY376	11		62.5	15.6	1.5
TSPY555	11		62.5	15.6	2.0
TSPY682	11		62.5	15.6	1.5
TSPY711	11	*aph(3')-III*	>2000	15.6	3.0
TSPY784	11		62.5	31.3	1.5
TSPY942	11		62.5	15.6	2.0
TSPY1115[2]	11	*aph(3')-III*	-	-	1.0
TSPY1165	11	*aph(3')-III*	>2000	15.6	2.0
TSPY1172	11		62.5	15.6	1.5
TSPY1239	11		62.5	15.6	3.0

Abbreviations: aminoglycoside (AG)

[1] Values represent MICs (ug/mL). MICs to kanamycin and streptomycin determined by broth microdilution and to gentamicin determined using Etest as described in Methods.

[2] TSPY1115 growth defective in liquid medium.

TABLE S7. Minimal inhibitory concentrations (MICs) to erythromycin and clindamycin for a subset of *emm77* GAS from Houston.

Strain	*emm* Type	Resistance Gene(s)	Erythromycin[1]	Clindamycin
TSPY165	77	*tet(M)*	0.016	0.032
TSPY311	77	*erm(A), tet(O)*	0.5	0.047
TSPY377	77	*erm(A), tet(O)*	1.0	0.047
TSPY453	77	*erm(A)*[2], *tet(O)*	2.0	>256
TSPY477	77	*tet(O)*	0.023	0.064

[1] Values represent MICs (ug/mL) determined using Etest as described in Methods.

[2] Upstream regulatory region of TSPY453 *erm(A)* contains two point mutations as described in text and Figure S4.

SUPPORTING FIGURES

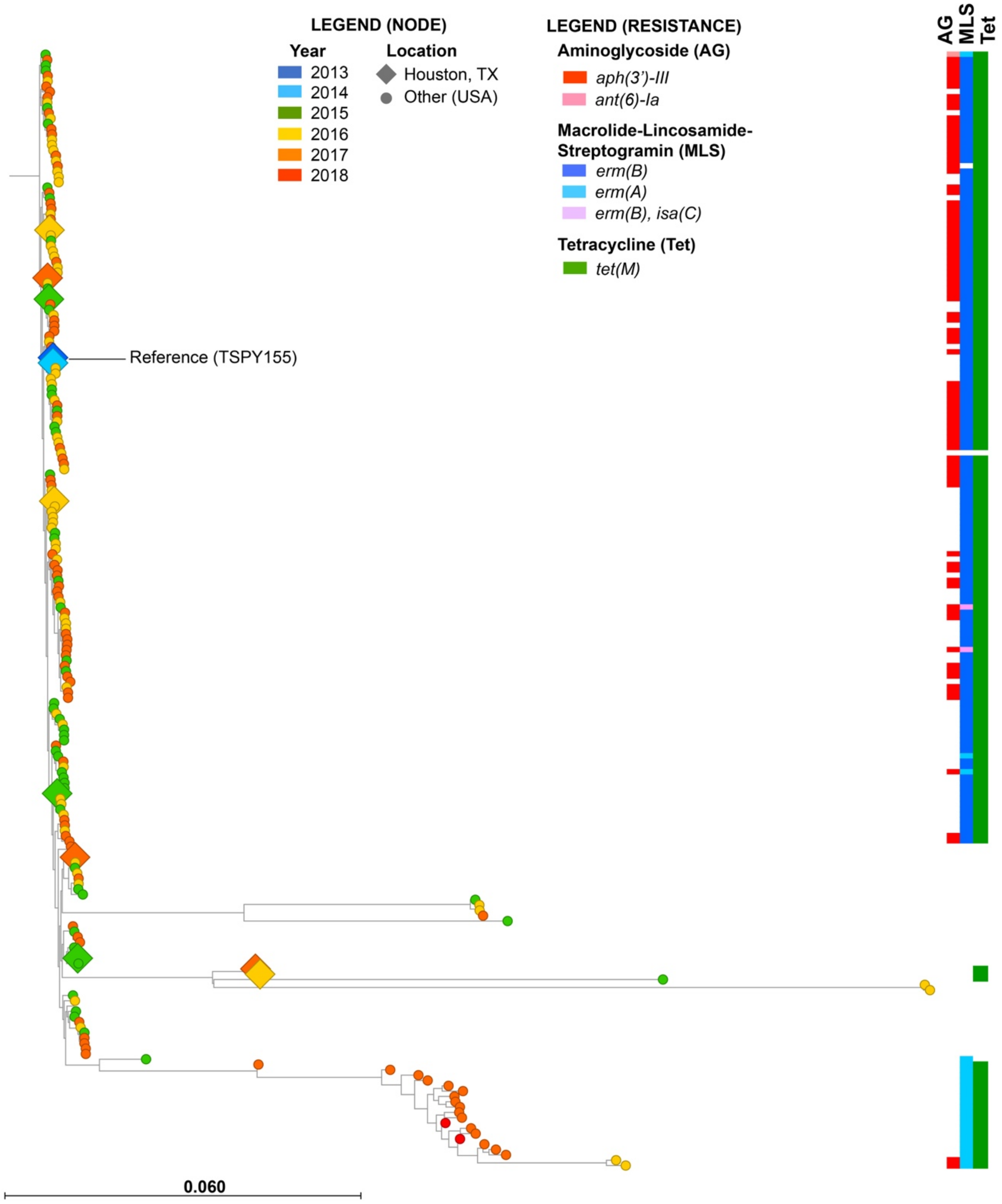

FIGURE S1. Phylogenetic reconstruction using 11,540 core biallelic SNP loci of *emm11* GAS from the Houston (n=10) and CDC ABCs (n=199) relative to the reference TSPY155. Node

color and shape indicate year of isolation and geographic location, respectively (node legend). Resistance genotype for aminoglycosides (AG), macrolide-lincosamide-streptogramin (MLS), and tetracycline (Tet) shown on right and colored by allele (resistance legend).

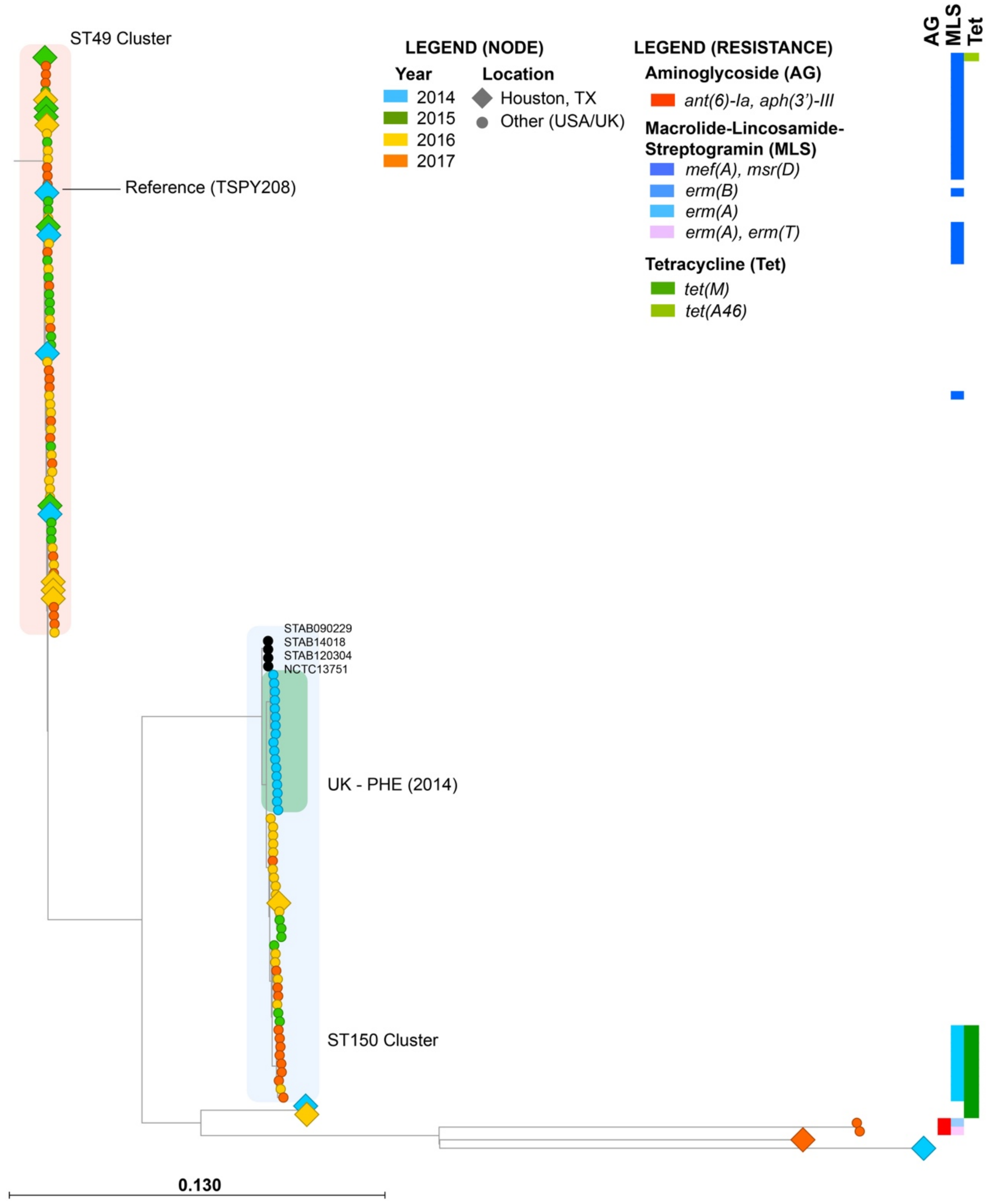

FIGURE S2. Phylogenetic reconstruction using 12,568 core biallelic SNP loci of *emm75* GAS strains from the Houston (n=16), CDC ABCs (n=88) and the UK (n=17) relative to the reference TSPY208. Completed reference genomes NCTC13751, STAB14018, STAB090229, and

STAB120304 are labeled. The two distinct multi-locus sequence type (MLST) clusters, ST49 and ST150, are shaded in red and blue, respectively. The *emm75* strains from the UK are shaded in green. Nodes and resistance genotype are depicted as in Figure S1.

FIGURE S3.

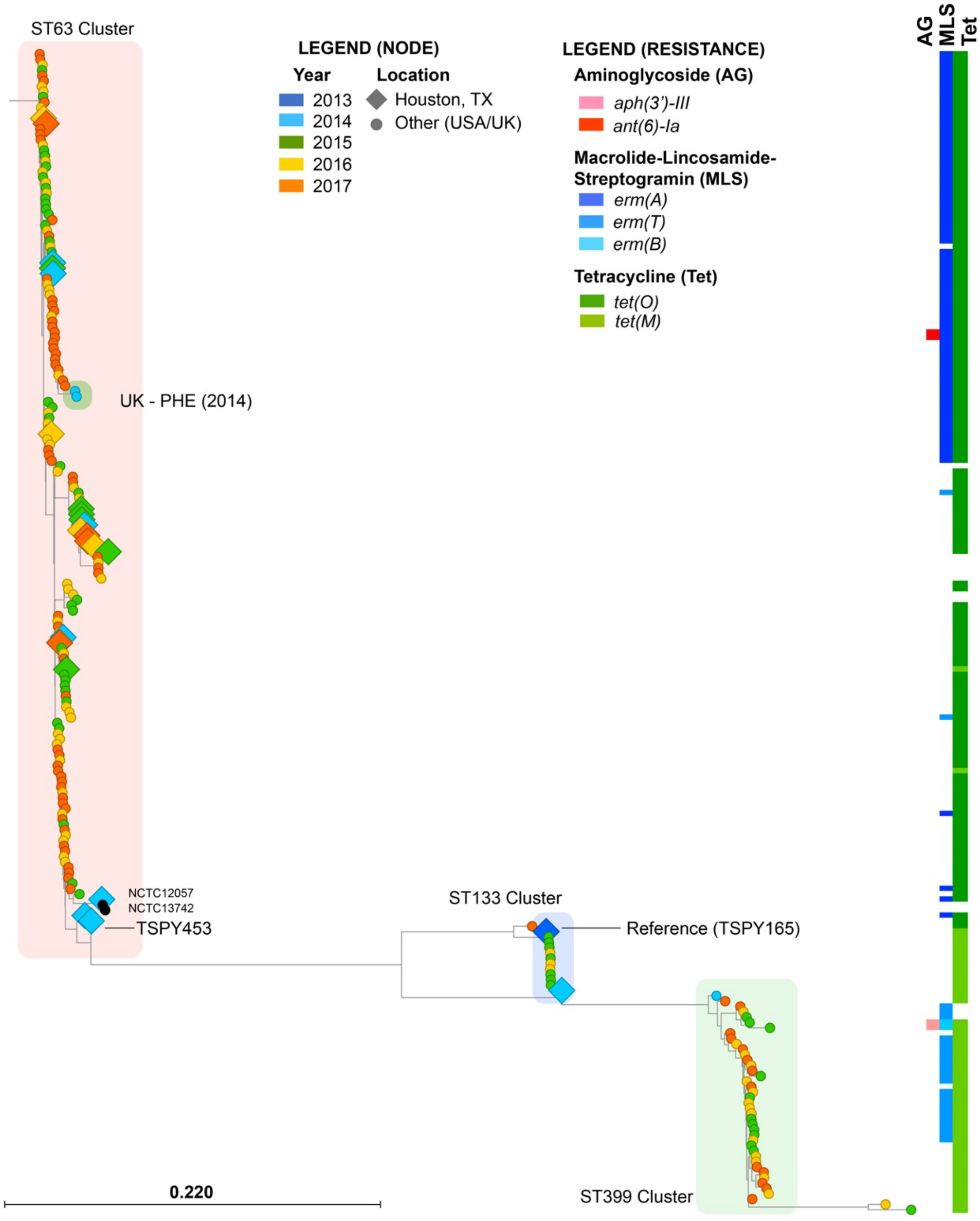

FIGURE S3. Phylogenetic reconstruction using 35,685 core biallelic SNP loci of *emm77* GAS strains from the Houston (n=21), CDC ABCs (n=189) and UK (n=3) relative to the reference

TSPY165. Completed reference genomes NCTC12057, NCGTC13742, and TSPY453 are labeled. Three distinct multi-locus sequence type (MLST) clusters are shaded and labeled. The *emm77* strains from the UK are shaded in green. Nodes and resistance genotypes are depicted as in Figure S1.

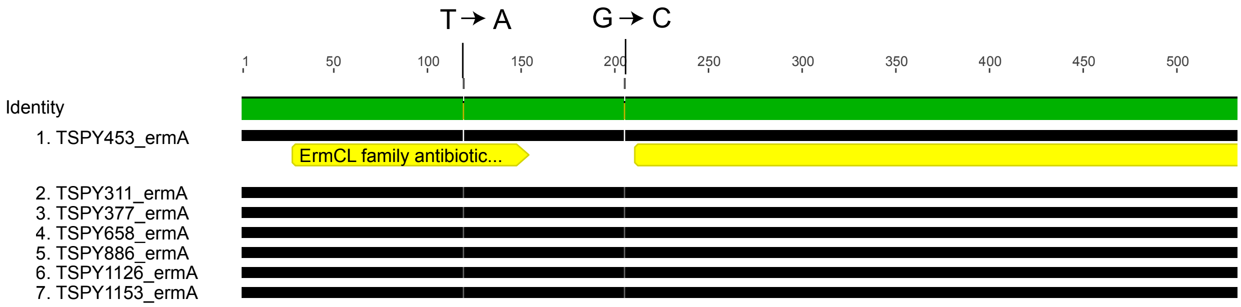

FIGURE S4. Nucleotide alignment of *erm(A)* upstream sequences in *emm77* GAS strains from Houston. Shown are sequences of strains subjected to MIC determinations in Table S6. Nucleotide changes in TSPY453 are indicated above the alignment. Yellow bars indicate the upstream leader peptide and coding sequence of *erm(A)* in TSPY453.

REFERENCES

1. **Koren, S., B. P. Walenz, K. Berlin, J. R. Miller, N. H. Bergman, and A. M. Phillippy.** 2017. Canu: scalable and accurate long-read assembly via adaptive k-mer weighting and repeat separation. *Genome Res.* **27:**722-736.
2. **Wick, R. R., L. M. Judd, C. L. Gorrie, and K. E. Holt.** 2017. Unicycler: Resolving bacterial genome assemblies from short and long sequencing reads. *PLoS Comput Biol.* **13:**e1005595.
3. **Chochua, S., B. J. Metcalf, Z. Li, J. Rivers, S. Mathis, D. Jackson, R. E. Gertz, Jr., V. Srinivasan, R. Lynfield, C. Van Beneden, L. McGee, and B. Beall.** 2017. Population and Whole Genome Sequence Based Characterization of Invasive Group A Streptococci Recovered in the United States during 2015. *MBio.* **8**.
4. **Chalker, V., A. Jironkin, J. Coelho, A. Al-Shahib, S. Platt, G. Kapatai, R. Daniel, C. Dhami, M. Laranjeira, T. Chambers, R. Guy, T. Lamagni, T. Harrison, M. Chand, A. P. Johnson, A. Underwood, and T. Scarlet Fever Incident Management.** 2017. Genome analysis following a national increase in Scarlet Fever in England 2014. *BMC Genomics.* **18:**224.
5. **Bolger, A. M., M. Lohse, and B. Usadel.** 2014. Trimmomatic: a flexible trimmer for Illumina sequence data. *Bioinformatics.* **30:**2114-20.
6. **Liu, Y., J. Schroder, and B. Schmidt.** 2013. Musket: a multistage k-mer spectrum-based error corrector for Illumina sequence data. *Bioinformatics.* **29:**308-15.
7. **Garrison, E., and G. Marth.** 2012. Haplotype-based variant detection from short-read sequencing. *arXiv preprint.*
8. **Price, M. N., P. S. Dehal, and A. P. Arkin.** 2010. FastTree 2--approximately maximum-likelihood trees for large alignments. *PLoS One.* **5:**e9490.
9. **Bankevich, A., S. Nurk, D. Antipov, A. A. Gurevich, M. Dvorkin, A. S. Kulikov, V. M. Lesin, S. I. Nikolenko, S. Pham, A. D. Prjibelski, A. V. Pyshkin, A. V. Sirotkin, N.

Vyahhi, G. Tesler, M. A. Alekseyev, and P. A. Pevzner. 2012. SPAdes: a new genome assembly algorithm and its applications to single-cell sequencing. *J Comput Biol.* **19:**455-77.

10. **Sullivan, M. J., N. K. Petty, and S. A. Beatson.** 2011. Easyfig: a genome comparison visualizer. *Bioinformatics.* **27:**1009-10.

11. **Arndt, D., J. R. Grant, A. Marcu, T. Sajed, A. Pon, Y. Liang, and D. S. Wishart.** 2016. PHASTER: a better, faster version of the PHAST phage search tool. *Nucleic Acids Res.* **44:**W16-21.

12. **Siguier, P., J. Perochon, L. Lestrade, J. Mahillon, and M. Chandler.** 2006. ISfinder: the reference centre for bacterial insertion sequences. *Nucleic Acids Res.* **34:**D32-6.

13. **Bertelli, C., M. R. Laird, K. P. Williams, G. Simon Fraser University Research Computing, B. Y. Lau, G. Hoad, G. L. Winsor, and F. S. L. Brinkman.** 2017. IslandViewer 4: expanded prediction of genomic islands for larger-scale datasets. *Nucleic Acids Res.* **45:**W30-W35.

14. **Liu, M., X. Li, Y. Xie, D. Bi, J. Sun, J. Li, C. Tai, Z. Deng, and H. Y. Ou.** 2019. ICEberg 2.0: an updated database of bacterial integrative and conjugative elements. *Nucleic Acids Res.* **47:**D660-D665.
